# Prosaposin in CNS health and disease, metabolic stress and exercise adaptation

**DOI:** 10.1007/s00109-026-02643-3

**Published:** 2026-02-02

**Authors:** Hash Brown Taha, Shirley Zhu, Eric Wang

**Affiliations:** 1 Washington University School of Medicine in St. Louis, St. Louis, MO USA; 2 Department of Biochemistry and Biomedical Sciences, McMaster University, Hamilton, ON Canada

**Keywords:** Alzheimer’s disease, Parkinson’s disease, Alpha-synuclein, Amyloid beta, Exerkine, Metabolism

## Abstract

Prosaposin (PSAP), a highly conserved lysosomal protein and precursor of saposins A–D, has emerged as a key regulator of cellular and central nervous system (CNS) homeostasis. Disrupted PSAP trafficking may lead to amyloid protein aggregation with implications for neurodegenerative diseases. In Alzheimer’s disease (AD) and Parkinson’s disease (PD), PSAP shows altered expression patterns and pathological co-localization with amyloid aggregates. *PSAP* variants are linked to multiple neurodegenerative diseases, including synucleinopathies, Gaucher’s disease, and metachromatic leukodystrophy. Its levels are elevated in blood and cerebrospinal fluid in some individuals with AD or PD and are upregulated by stress conditions such as nerve injury and cold adaptation, but not by exercise. Prosaptides, short peptides derived from PSAP, show protective effects in models of oxidative stress, CNS injury, and metabolic disorders. Pharmacological stabilization of PSAP interactions with progranulin has shown promise in neurodegenerative disease models. These findings suggest PSAP plays an important role in maintaining brain health and may hold therapeutic potential. Here, we provide a comprehensive overview of PSAP’s role in CNS health and disease, metabolic stress, and exercise adaptation.

**Supplementary Information:**

The online version contains supplementary material available at 10.1007/s00109-026-02643-3.

## Introduction

The lysosome has recently emerged as a central organelle in metabolic sensing and trans-cellular signaling, playing a paramount role in the regulation of cellular homeostasis and longevity. Beyond its traditional function in degradation, the lysosome integrates nutrient, stress, and energy cues to coordinate intracellular and intercellular responses [1]. Prosaposin (PSAP), a key lysosomal protein, is increasingly recognized within this signaling network [2] and serves as the precursor for the sphingolipid hydrolase activator proteins known as saposins. PSAP is highly conserved across species, especially vertebrates [3–5].

The first description of a sphingolipid activator protein subunit dates back to 1964, when Mehl and Jatzkewitz discovered saposin B (originally termed “sphingolipid activator protein 1”) as a heat-stable cofactor enhancing arylsulfatase A (ARSA) activity [6]. In 1971, Ho and O’Brien identified saposin C (originally termed “factor P” and later “sphingolipid activator protein 2”) from the spleen tissue of a 12-year-old female with type 3 Gaucher’s disease (GD) following splenectomy. Saposin C was heat-resistant and capable of restoring mutant glucocerebrosidase (GCase) activity *in vitro* [7]*.* Cloning studies in the late 1980s revealed that both saposins B and C are derived from a common 73 kDa glycoprotein precursor, later named PSAP [8–11]. Subsequent analyses uncovered two additional homologous domains within PSAP corresponding to saposins A and D [12–14]. Despite PSAP’s early demonstrated therapeutic and neurotrophic potential, PSAP remained largely overlooked following the death of John S O’Brien, who played a seminal role in advancing research on its neurotrophic functions. However, recent studies have reignited interest in PSAP’s potential in central nervous system (CNS) health, aging and disease, and metabolic stress, and exercise adaptation.

Disruption of PSAP trafficking to the lysosome leads to increased PSAP levels in circulation and promotes protein aggregation [15]. In neurodegenerative disorders such as Alzheimer’s disease (AD), PSAP co-localizes and positively correlates with amyloid beta (Aβ) plaques [16, 17]; in Parkinson’s disease (PD), PSAP is increased in dopaminergic (DA) neurons in the substantia nigra and associates with motor symptom dysfunction severity [18]; in ALS autopsy cases, PSAP localizes to Bunina bodies and lipofuscin aggregates within spinal cord motor neurons, with partial colocalization with cystatin C and decreased diffuse cytoplasmic expression in surviving neurons [19]. PSAP levels and gene expression are highly regulated throughout CNS neurodevelopment [20–22]. Genetic variation in *PSAP* is associated with several neurodegenerative disorders including GD [23], metachromatic leukodystrophy [24], and PD [25]. PSAP can be detected in multiple biofluids including blood [26], and its secretion is enhanced under conditions of cellular stress, including sciatic and spinal nerve transection [27], and cold adaptation [28], but not exercise [28, 29]. A few reports suggest that PSAP levels may be increased in the blood and CSF of persons with PD [18, 30] and AD [31, 32].

Full-length PSAP and short peptides derived from saposin C, known as “prosaptides,” improve in vitro and in vivo models’ outcomes of oxidative stress [33, 34], ischemia [35–41], CNS injury [27, 42–45], diabetes [46–49], hyperalgesia/allodynia [50–52], AD [53], and PD [18, 54–57]. Pharmaceuticals that target and stabilize PSAP’s interactions with progranulin (PRGN), an important molecule for brain homeostasis [58], have shown promise for progressive supranuclear palsy (PSP) [59]. Taken together, these findings portray PSAP as a paramount molecule in CNS health and disease.

In this review, we examine PSAP’s biological and physioological functions, its genetic associations with neurodegenerative disorders, its positive and negative roles in brain homeostasis, response to metabolic stress and exercise, and potential as a biomarker and a neurotherapeutic molecule.

## PSAP: structure, function, and secretory pathways

**Fig. 1:**
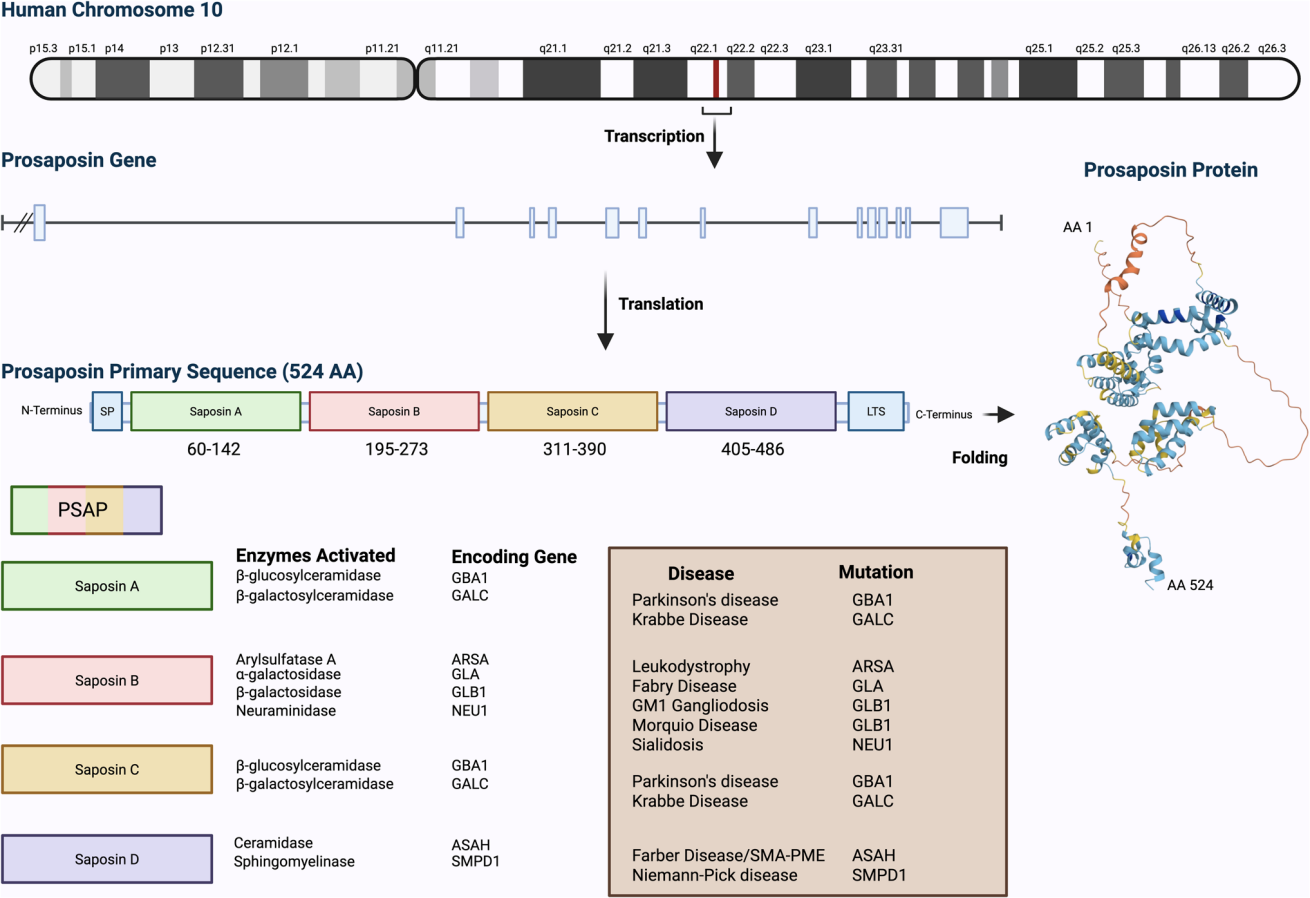
Prosaposin (PSAP) structure and genomic organization of the human *PSAP* gene and protein, and functional domains of saposins A–D. This schematic illustrates the chromosomal location, exon–intron structure, primary sequence, and domain organization of the human *PSAP* gene and encoded prosaposin protein. The *PSAP* gene is located on chromosome 10q22.1 and undergoes transcription and translation to produce a 524-amino-acid precursor protein. PSAP contains a signal peptide (SP) at the N-terminus followed by four conserved domains: saposin A (residues 60–142), saposin B (195–273), saposin C (311–390), and saposin D (405–486). A lysosomal targeting sequence (LTS) precedes the C-terminal end. After proteolytic cleavage in the lysosome, each saposin domain activates specific sphingolipid-degrading enzymes. Below the domain map, each saposin is shown with its associated target enzymes and encoding genes. The diseases associated with mutations in the corresponding enzymes are also listed, including Parkinson’s disease, Krabbe disease, Fabry disease, and others. On the right, the predicted tertiary structure of human prosaposin (based on AlphaFold https://alphafoldserver.com/) is depicted, showing the protein from N- to C-terminus (AA 1–524), highlighting folding and domain organization. Colors within the tertiary structure represent per-residue confidence with blue depicting very high confidence (pLDDT > 90), cyan depicting moderate confidence (pLDDT 70–90), yellow depicting low confidence (pLDDT 50–70), and orange/red depicting very low confidence (pLDDT < 50). Abbreviations: AA, amino acid; ARSA, arylsulfatase A; CNS, central nervous system; GBA1, β-glucocerebrosidase; GALC, β-galactosylceramidase; GLA, α-galactosidase; GLB1, β-galactosidase; LTS, lysosomal targeting sequence; NEU1, neuraminidase 1; SMPD1, sphingomyelin phosphodiesterase 1; ASAH, acid ceramidase; SP, signal peptide. Created with BioRender.com

The human *PSAP* gene is located on chromosome 10q22.1, consists of 14 exons and 13 introns, and encodes the full-length (524 aa) PSAP glycoprotein, including a signal peptide, four homologous saposin domains A–D, and a 17 amino acid lysosomal trafficking signal [60–62] (Fig. 1). Each of the four saposins derived from PSAP through posttranslational modifications comprises 79–82 amino acids and contains six regularly spaced cysteines, two prolines, and one glycosylation site (except two in saposin A). Due to their high heat stability, dense disulfide bonding, and resistance to proteolysis, saposins are considered compact, rigid structures. They predominantly adopt an α-helical conformation, particularly at an acidic pH (~ 4.5), which aligns with the optimal environment for lysosomal hydrolases [63].

Saposins serve essential roles as lipid-binding cofactors that facilitate the lysosomal degradation of sphingolipids by specific hydrolases, with each saposin interacting with distinct enzymes [63]. Saposin A activates glucocerebrosidase (GBA1) [14] and galactosylceramidase (GALC) [64], supporting the breakdown of glucocerebrosides and galactocerebrosides, respectively. Saposin B aids ARSA [65], α-galactosidase (GLA) [66], β-galactosidase 1 (GLB1) [67], and neuraminidase 1 (NEU1) [68], playing roles in the degradation of sulfatides, globotriaosylceramide, GM1 ganglioside, and sialic acid-containing glycoproteins, respectively. Saposin C, similar to saposin A, is primarily associated with GBA1 [69] and GALC [67]. Saposin D, though less characterized, activates acid ceramidase (ASAH1) [69] and sphingomyelin phosphodiesterase 1 (SMPD1) [13], contributing to ceramide and sphingomyelin hydrolysis.

**Fig. 2:**
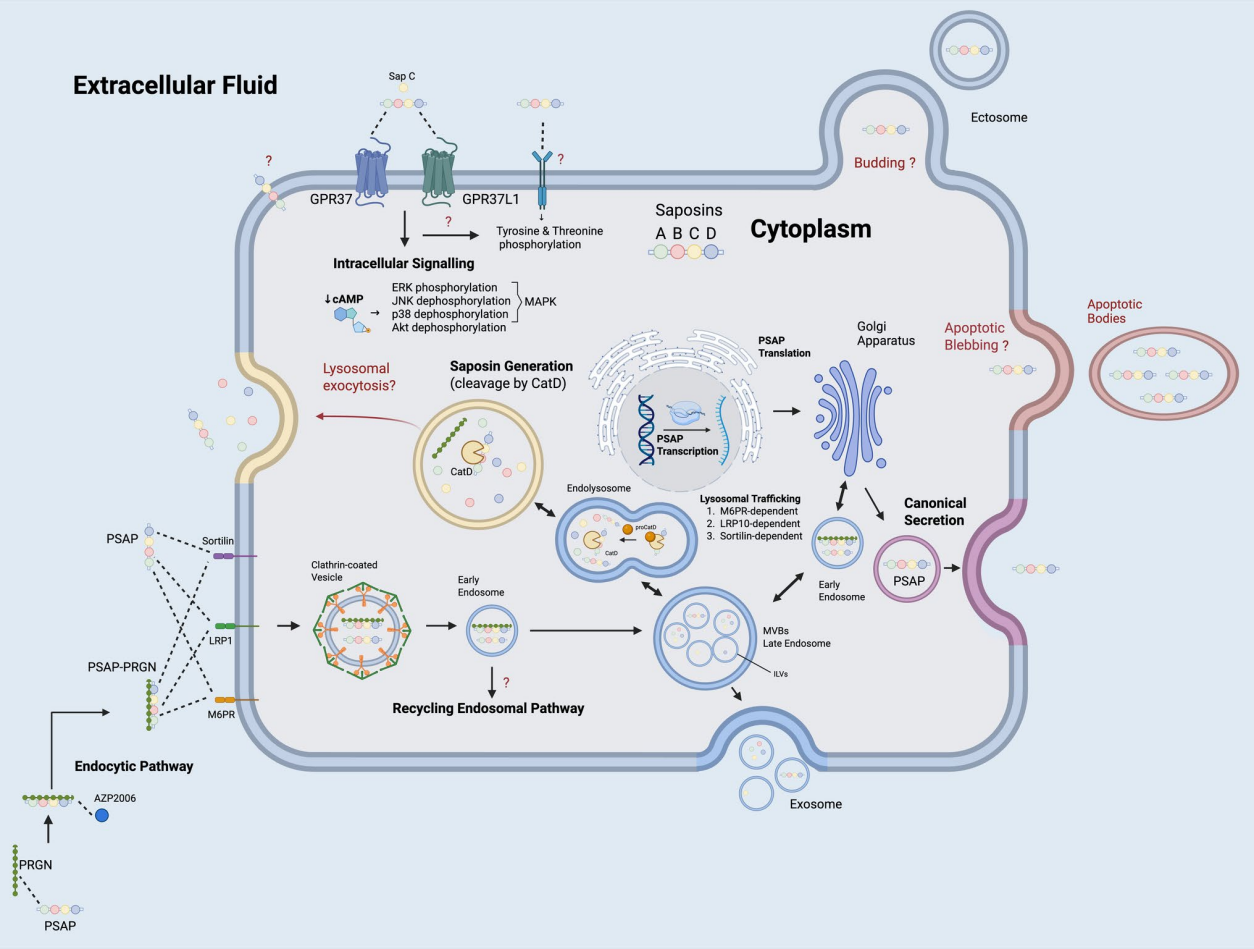
Cellular pathways of prosaposin (PSAP) trafficking, processing, secretion, and intracellular signaling. This schematic illustrates the intracellular trafficking, processing, and secretion routes of prosaposin (PSAP) and its cleavage products, saposins A–D, in mammalian cells. Following *PSAP* transcription and translation in the cytoplasm, PSAP is transported through the Golgi apparatus and targeted to lysosomes via three major receptor-mediated pathways: the mannose-6-phosphate receptor (M6PR), the LDL receptor-related protein 1 (LRP1), and sortilin. Within lysosomes, PSAP is cleaved by cathepsin D (CatD) to generate the four functional saposin domains. These saposins are involved in glycosphingolipid degradation. PSAP and saposins can be secreted via several routes, including canonical secretion through the Golgi–endosome pathway and non-canonical routes such as exosome release from multivesicular bodies (MVBs), ectosome budding, and apoptotic blebbing. PSAP and saposin C can also be found in the extracellular space, where they may interact with membrane-bound receptors such as GPR37 and GPR37L1, initiating downstream signaling cascades involving reduced cAMP and modulation of MAPK, ERK, JNK, and Akt phosphorylation. Additionally, PSAP or PSAP-PRGN complexes can undergo clathrin-mediated endocytosis via interactions with sortilin, LRP1, or M6P receptors, followed by trafficking into early endosomes or recycling pathways. Pharmacological agents such as AZP2006 may affect this internalization process. Several aspects of these pathways, including the potential for lysosomal exocytosis and the roles of ectosome and apoptotic body formation, remain incompletely defined and are denoted by question marks. The dashed lines refer to protein–protein interactions. Abbreviations: CatD, cathepsin D; ERK, extracellular signal-regulated kinase; GPR37, G protein-coupled receptor 37; GPR37L1, G protein-coupled receptor 37-like 1; ILV, intraluminal vesicle; JNK, c-jun N-terminal kinase; LRP1, low-density lipoprotein receptor-related protein 1; MAPK, mitogen-activated protein kinase; M6PR, mannose-6-phosphate receptor; MVB, multivesicular body; PRGN, progranulin; SAP A–D, saposins A, B, C, and D. Created with BioRender.com

PSAP follows one of three major intracellular pathways after being routed to the Golgi apparatus (Fig. 2). In the canonical lysosomal route, PSAP can be trafficked to the lysosome in a sortilin-dependent [70, 71] or sortilin-independent manner alone or as a PSAP-PRGN complex by binding to the cation-independent mannose 6-phosphate receptor (CI-M6PR) [72] or lipoprotein receptor related protein (LRP) 10 [73]. In the lysosome, cathepsin D proteolytically cleaves PSAP into four functional saposins: A, B, C, and D [74]. In the secretory route, PSAP can be directed to constitutive secretory vesicles for release into the extracellular environment. PSAP-deficient mice and patients accumulate multivesicular bodies in brain and skin cells [75–77], and PSAP has been detected in proteomic analysis of extracellular vesicles (EVs) isolated from various cells and biofluids [78–82] with functional roles in macrophage immune activation [83], strongly suggesting it may also be released through nonclassical mechanisms such as EVs. Given that PSAP is actively trafficked to lysosomes, it necessarily transits through late endosomes and multivesicular bodies, supporting the notion that its EV release likely occurs via exosome-mediated export; while direct ectosome budding from the plasma membrane or inclusion in apoptotic bodies is possible, these routes are likely less predominant.

Extracellularly, PSAP acts mainly on the G-protein coupled receptors 37 and 37-like 1 (GPR37 and GPR37L1) leading to intracellular signaling cascades [84, 85], to MAPK/ERK and Akt activation [86–88] or to be internalized through the endocytic pathway via CI-M6PR or LRP1 receptors [72, 89] (Fig. 2). Some studies reported PSAP and saposin C phosphorylation of tyrosine and threonine proteins, suggesting they may directly or indirectly lead to activation of tyrosine kinase receptors [90, 91]. PSAP may also localize to the plasma membrane, but its functions in this compartment remain poorly characterized [92].

## PSAP genetic variants link to neurodegeneration

**Fig. 3:**
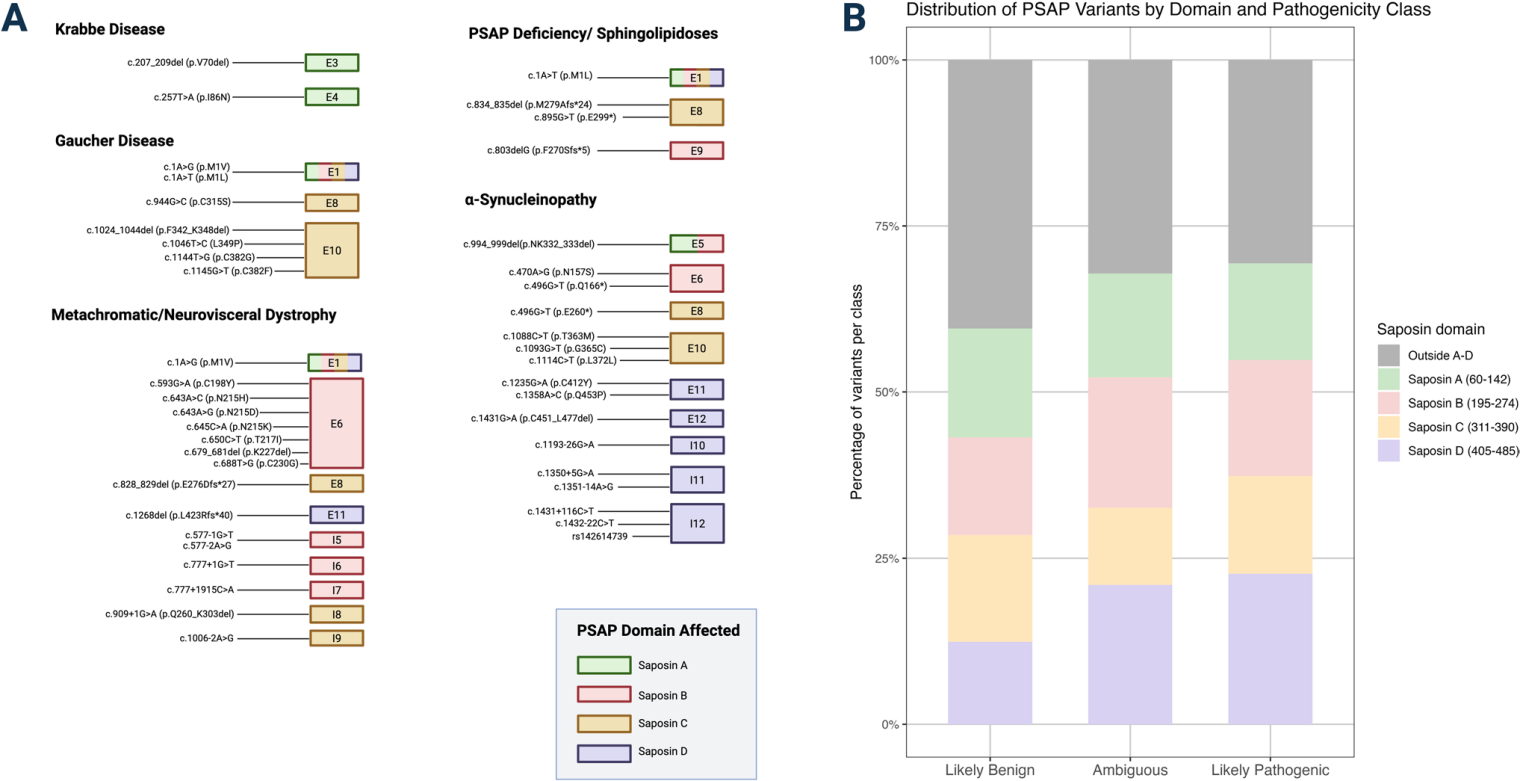
Disease-associated prosaposin (PSAP) variants and distribution by saposin domain and pathogenicity classification. **A** Disease-linked PSAP variants are mapped to specific saposin domains across Krabbe disease, Gaucher disease, metachromatic leukodystrophy (or neurovisceral dystrophy), α-synucleinopathies, and other sphingolipidoses. Genetic variants span exons encoding saposins A–D, with several variants recurring across disease categories. The domain affected (color-coded) is shown beside each mutation. For full variant details including genomic position, amino acid change, and supporting references, see Table 1. **B** Stacked bar plot shows the relative distribution of PSAP variants by saposin domain and predicted AlphaMissense pathogenicity class (likely benign, ambiguous, likely pathogenic). Variants affecting saposin D and saposin C are more frequently categorized as likely pathogenic, while those outside the saposin domains are predominantly benign. Colors indicate domain assignment: green (saposin A), pink (saposin B), yellow (saposin C), purple (saposin D), and gray (outside A–D). Created with BioRender.com

At least 34 genetic variants have been identified in the *PSAP* gene (see Table 1 for details), several of which are pathogenic mutations associated with lysosomal storage and neurodegenerative disorders (Fig. 3A). Researchers first identified saposin C in a patient with GD [7], and subsequent studies confirmed that a few individuals carrying genetic variants in saposin C, an essential coactivator of GCase, may develop clinical manifestation of Gaucher’s-like disease [23, 93], although to a rarer extent than those with biallelic *GBA1* genetic variants. Additionally, genetic variation in the saposin A region have been associated with Krabbe disease [94, 95], saposin B with metachromatic leukodystrophy [96, 97], and saposin D potentially with PD [98].

**Table 1:** Summary of PSAP mutations across diseases. Mutation information was confirmed using https://mutalyzer.nl/, https://www.ncbi.nlm.nih.gov/clinvar/, and https://www.ncbi.nlm.nih.gov/snp/ based on the canonical PSAP mRNA transcript NM_002778.4

PMID	Author	Year	Country	Disease	Method	Domain	Type (on hg38 Genome)	Specific mutation (ClinVar)	Age	Sex	Ancestry	Tissue
Krabbe												
15773042	Spiegel et al.	2005	Israel	Krabbe	Mutations were identified using RT-PCR followed by SSCP analysis, sequencing, and confirmation by genomic PCR with Aat II restriction digestion	Saposin A	Exon 3	c.207_209del (p.V70del)	6 months at clinical presentation	Female	Arab	Patient fibroblasts; parental lymphoblasts
31439510	Calderwood et al.	2020	USA	Krabbe	Whole exome sequencing with targeted analysis of PSAP; confirmed by biparental inheritance	Saposin A	Exon 4	c.257 T > A (p.I86N)	6 months at clinical presentation	Male	Indian	Leukocytes
Gaucher												
5288260	Ho & O’Brien	1971	USA	Gaucher	Saposin C enzyme activity in spleen of patient	Saposin C	NA	NA	12 years	Female	NA	Spleen
3675870	Christomanou et al.	1987	Germany	Gaucher	No sequencing: examination of spleen tissue using protein purification, tryptic digestion, and Edman degradation to determine the N-terminal amino acid sequence of saposin C	Saposin C	NA	NA	60 years	Female	NA	Spleen
2514102	Harzer et al.	1989	Germany	Gaucher	No sequencing: examination of skin fibroblasts, leukocytes, amniotic fluid cells, liver, nerve, and muscle biopsies using biochemical assays, metabolic labeling, immunoblotting, and ultrastructural analysis revealed absence of Saposin C	Saposin C	NA	NA	NA	Male	NA	Multiple
2060627	Schnabel et al.	1991	Germany	Gaucher	Northern blot for saposin C transcript; Sanger sequencing	Saposin C	Exon 10	c.1145G > T (p.C382F)	NA	Female	Caucasian	Fibroblasts
8460394	Rafi et al.	1993	USA	Gaucher	PCR amplification of genomic DNA and cDNA, Northern blotting and Sanger sequencing	Saposin C	Exon 10	c.1144 T > G (p.C382G)	NA	NA	Caucasian	Patient spleen; parental fibroblasts
9930900	Pàmpols et al.	1999	Spain	Gaucher	No sequencing; examination through autopsy pathology, biochemical lipid profiling (TLC, GC), and histological analysis	Saposin C	NA	NA	8 years (onset)	Male	NA	Brain, spleen, liver, lymph nodes, bone marrow, thymus, lungs, adrenal glands and large intestine
17919309	Tylki-Szymańska et al.	2007	Poland	Gaucher	Exons amplified from genomic DNA and products sequenced with CEQ 2000 Beckman Coulter sequencer	Saposin C	Exon 1; Exon 10	c.1A > T (p.M1L); c.1046 T > C (p.L349P)	35 years	Male	NA	Peripheral blood; fibroblasts
17919309	Tylki-Szymańska et al.	2007	Poland	Gaucher		Saposin C	Exon 1; Exon 10	c.1A > T (p.M1L); c.1046 T > C (p.L349P)	~ 29 years	Female	NA	Peripheral blood; fibroblasts
20484222	Vaccaro et al.	2010	Italy	Gaucher	NA; probably Sanger sequencing	Saposin C	Exon 1; Exon 10	c.1A > T (p.M1L); c.1046 T > C (p.L349P)	NA	Male	Polish	Fibroblasts
20484222	Vaccaro et al.	2010	Italy	Gaucher	NA; probably Sanger sequencing	Saposin C	Exon 1; Exon 10	c.1A > T (p.M1L); c.1046 T > C (p.L349P)	NA	Female	Polish	Fibroblasts
20484222	Vaccaro et al.	2010	Italy	Gaucher	NA; probably Sanger sequencing	Saposin C	Exon 1; Exon 8	c.1A > G (p.M1V); c.944G > C (p.C315S)	NA	NA	French	Fibroblasts
20484222	Vaccaro et al.	2010	Italy	Gaucher	NA; probably Sanger sequencing	Saposin C	Exon 10	c.1024_1044del (p.F342_K348del)	5 years	NA	Indian	Fibroblasts
Metachromatic/neurovisceral dystrophy												
2320574	Kretz et al.	1990	USA	Metachromatic leukodystrophy	Sanger Sequencing	Saposin B	Exon 6	c.650C > T (p.T217I)	NA	Female	NA	Lymphoblasts
1683286	Schlote et al.	1991	Germany	Metachromatic leukodystrophy	No sequencing: Examination using biochemical assays, thin-layer chromatography, immunodiffusion, and electron microscopy	Saposin B	NA	NA	7 years	Male	Middle Eastern	Fibroblasts; leukocytes; urine; rectal biopsy
1689485	Zhang et al.	1990	USA	Metachromatic leukodystrophy	Sanger sequencing: PCR amplification of full-length cDNA and genomic DNA from cultured skin fibroblasts and transformed lymphoblasts, subcloned into M13 vectors and sequenced using the Sanger method	Saposin B	Intron 7	c.777 + 1915 C > A	NA	Female	NA	Fibroblasts; lymphoblasts
2302219	Rafi et al.	1990	USA	Metachromatic leukodystrophy	Amplification of cDNA	Saposin B	Exon 6	c.650C > T (p.T217I)	NA	Female	NA	Fibroblasts
8554069	Henseler et al.	1996	Germany	Metachromatic leukodystrophy	Sanger Sequencing	Saposin B	Intron 7	c.777 + 1G > T	< 2 years age	Male	Turkish	Fibroblasts
9672525	Landrieu et al.	1998	France	Metachromatic leukodystrophy	No sequencing: examination of saposin B protein levels by immunoprecipitation, electrophoresis, and autoradiography	Saposin B	NA	NA	25 months (onset)	Male	NA	Fibroblasts
10196694	Regis et al.	1999	Italy	Metachromatic leukodystrophy	Sanger sequencing	Saposin B	Exon 6	c.645C > A (p.N215K)	3–5 years	Male	NA	Fibroblasts
10682309	Wrobe et al.	2000	Germany	Metachromatic leukodystrophy	Sanger sequencing	Saposin B	Exon 6	c.643A > C (p.N215H)	4 years	Female	Spanish	Fibroblasts
17616409	Deconinck et al.	2008	Belgium	Metachromatic leukodystrophy	NA	Saposin B	Exon 1; Exon 6	c.1A > G (p.M1V); c.645C > A (p.N215K)	2 years	Female	Italian	NA
18693274	Grossi et al.	2008	Italy	Metachromatic leukodystrophy	Sanger sequencing	Saposin B	Exon 6; Intron 5	Three patients: c.645C > A (p.N215K); One patient: c.577-1G > T	NA	NA	NA	Not specified: fibroblasts; lymphoblasts; peripheral blood
19267410	Kuchar et al.	2009	Czech Republic	Neurovisceral dystrophy	Genomic DNA and cDNA sequencing (RT-PCR); splice site mutation analysis	Saposin C	Intron 9	c.1006-2A > G	NA	Male	NA	Cultured fibroblasts; leukocytes
19267410	Kuchar et al.	2009	Czech Republic	Metachromatic leukodystrophy-like disorder (normal ARSA activity)	Genomic DNA and cDNA sequencing (RT-PCR); splice site mutation analysis	Saposin B; saposin C	Intron 5; Exon 8	c.577-2A > G and c.828_829del (p.E276Dfs*27)	NA	Male	NA	Cultured fibroblasts; leukocytes
24478108	Siri et al.	2014	Italy	Metachromatic leukodystrophy	Sanger sequencing	Between saposins B and C	Intron 8	c.909 + 1G > A (p.Q260_K303del)	27 months	Male	Moroccan	Fibroblasts
24478108	Siri et al.	2014	Italy	Metachromatic leukodystrophy	Sanger sequencing	Between saposins B and C	Intron 8	c.909 + 1G > A (p.Q260_K303del)	24 months	Male	Moroccan	Fibroblasts
30632081	Kolnikova et al.	2019	Slovakia	Metachromatic leukodystrophy	Sanger sequencing	Saposin B; saposin D	Exon 6; Exon 11	c.679_681del (p.K227del); c.1268del (p.L423Rfs*40)	4 years	Male	NA	Peripheral blood
31319425	Madaan et al.	2019	India	Metachromatic leukodystrophy	Genetic panel for leukodystrophy	Saposin B	Exon 6	c.679_681del (p.K227del)	3.5 years	Male	Indian	NA
37404680	Sheth et al.	2023	India	Metachromatic leukodystrophy	Targeted exome sequencing	Saposin B	Exon 6	c.688 T > G (p.C230G)	3 years	Male	Indian	Peripheral blood
37404680	Sheth et al.	2023	India	Metachromatic leukodystrophy	Targeted gene panel using single molecule molecular inversion probes; Sanger sequencing confirmed in proband	Saposin B	Exon 6	c.593G > A (p.C198Y)	19 years	Male	Indian	Peripheral blood
39,612,318	Li et al.	2024	China	Metachromatic leukodystrophy	Whole exome and Sanger sequencing	Saposin B	Exon 6	c.643A > G (p.N215D)	2.5 years	Female	NA	Peripheral blood
PSAP deficiency or sphingolipdosis												
1637339	Paton et al.	1992	Australia	PSAP deficiency	No sequencing: cerebroside sulfate and globotriaosylceramide turnover in patient-derived fibroblasts	Saposin B; Saposin C	NA	NA	NA	NA	NA	Multiple
11309366	Hulková et al.	2001	Czech Republic	Sphingolipidosis	Sanger sequencing	Saposin B	Exon 9	c.803del (p.F270Sfs*5)	0–3.5 months	Male	Slovakian	leukocytes; fibroblasts
11309366	Hulková et al.	2001	Czech Republic	Sphingolipidosis	Sanger sequencing using maternal cDNA	Saposin B	Exon 9	c.803del (p.F270Sfs*5)	neonatal death	Female	Slovakian	Maternal fibroblasts
15944902	Elleder et al.	2005	Czech Republic	PSAP deficiency	Sanger Sequencing	PSAP	Exon 1	c.1A > T (p.M1L)	Baby (NA)	Male	NA	Fibroblasts
26831127	Motta et al.	2016	Italy	PSAP deficiency	Variant screening by Losoplex (targeted resequencing panel)	Between saposins B and C	Exon 8	c.895G > T (p.V299L)	2 months (referral)	Female	Italian	Fibroblasts
26831127	Motta et al.	2016	Italy	PSAP deficiency	Sanger sequencing of PSAP coding sequence	Between saposins B and C	Exon 8	c.834_835del (p.M279Afs*24)	2 months (referral)	Female	Macedonian	Fibroblasts
α-Synucleinopathy												
30037697	Bencheikh et al.	2018	Canada	Parkinson’s disease (FCFR *n* = 544, NY = 579; Rubak et al. public dataset = 1167); NC (FCFR = 869, NY = 284; Rubak et al. public dataset = 1685)	Molecular inversion probes and Sanger sequencing	Saposin C	Exon 10	c.1088C > T (p.T363M; Rubak et al.public dataset); c.1093G > T (p.G365C; FCFR cohort + Rubak et al. public dataset); c.1114C > T (p.L372L; FCFR cohort);	See paper	See Paper	See Paper	Peripheral blood
32201884	Oji et al.	2020	Japan	Parkinson's disease	Sanger and whole-exome sequencing	Saposin D	Exon 11; Exon 11; Exon 12; Intron 10; Intron 11; Intron 11; Intron 12; Intron 12; Intron 13	c.1235G > A (p.C412Y); c.1358A > C (p.Q453P); c.1431G > A (p.C451_L477del); c.1193-26G > A; c.1350 + 5G > A; c.1351-14A > G; c.1431 + 116 C > T; c.1432-22C > T; c.1540-34C > T	Varies (see paper	(Varies see paper)	Japanese; Taiwanese	Peripheral blood
33197249	Lin et al.	2020	China	Parkinsons disease (*n* = 487); NC (*n* = 482)	Sanger sequencing	Saposin D	Intron 10; Intron 11; Intron 11; Intron 12; Intron 12; Intron 12	c.1193-26G > A; c.1350 + 5G > A; c.1351-14A > G; rs142614739; c.1431 + 116 C > T; c.1432-22C > T	PD: 60.2 ± 11.7; NC: 59.2 ± 13.1	PD male/female: 279/208; NC male/female: 263/219)	Asian	Peripheral blood
33402667	Aslam et al.	2021	Germany	Parkinson’s disease (*n* = 4)	Whole genome and Sanger sequencing	Intersaposin A–B region	Exon 6	c.470A > G (p.N157S)	49–80 (range)	Male	South Asian	Peripheral blood
34690151	Sosero et al.	2022	Canada	iRBD (*n* = 1113 tested, *n* = 3 with LoF mutations); NC (*n* = 2324)	Molecular inversion probes and Sanger sequencing	Intersaposin A–B region; saposin B; saposin C	Exon 5; Exon 6; Exon 8	c.496G > T (p.Q166*), *n* = 1 c.778C > T (p.E260*), *n* = 1 c.994_999del (p.NK332_333del), *n* = 1	75–79 years; 80–84 years; 60–64 years	All male	NA	NA

*PSAP* genetic variants have recently gained attention as a potential risk factor for α-synucleinopathy development. In isolated REM sleep behavior disorder (iRBD), a prodromal α-synucleinopathy characterized by dream-enactment behaviors and a high risk of conversion to PD, Lewy body dementia, and/or multiple system atrophy (MSA) [99], *PSAP* loss-of-function (LoF) variants have shown relevance. In a genetic study of 1113 persons with iRBD and 2324 controls, three LoF *PSAP* variants were identified exclusively in persons with iRBD, two of whom also carried *GBA1* variants [100]. An in silico structural analysis revealed that these *PSAP* LoF variants have a deleterious effect on saposin C (two stop codons, p.E166T* and p.Q260T*, and one p.N332_K333del), supporting a link between saposin C and α-synucleinopathy development. Although none of the three persons phenoconverted to an α-synucleinopathy at follow-up, all exhibited early motor and cognitive deficits, and each had a > 98% probability of prodromal PD. Notably, recent genome-wide association studies (GWAS) of individuals with iRBD and RBD with PD identified significant risk variants only in *SNCA*, *GBA1*, *TMEM175*, *INPP5F/BAG3*, and *SCARB2*. This absence of association with PSAP may be due to methodological limitations of GWAS, which are designed to detect common rather than rarer variants [101]. These findings suggest that while PSAP may not contribute to iRBD risk at the population level, it could play a meaningful role in a subset of individuals with a distinct rare-variant genetic architecture.

In one PD family, whole-genome sequencing identified a rare *PSAP* variant (p.N157S in the saposin B domain), particularly in the context of familial *GBA1* mutation carriers. When this *PSAP* variant was expressed in HEK293 cells, it led to lysosomal defects and increased α-synuclein aggregates [102]. However, another study fully sequenced *PSAP* in a larger, non-familial cohort (1123 PD, 1153 controls), and found no genetic association between *PSAP* and PD [103]. These differences could suggest that *PSAP* variants contribute only to a subset of individuals who go on to develop familial PD or that PSAP variants are risk factors for other α-synucleinopathies such as MSA or DLB in patients initially presenting with iRBD, although this remains speculative. Variation in the intronic regions of exons 10 to 14 (encoding saposin D) may be implicated in sporadic, familial, and early-onset PD cases [98, 104]. However, saposin D genetic variations in humans and their association with α-synucleinopathy remain debated [105].

Though no *PSAP* variants have been linked to FTLD, *PRGN* LoF mutations can cause FTLD [106]. PSAP associates closely with PRGN in the cell, and PSAP single nucleotide polymorphisms can strongly predict plasma PRGN levels. For example, *PSAP* deficiency leads to increased PRGN monomer expression, while its overexpression leads to increased PRGN oligomer expression [107]. Additionally, a gene–gene interaction analysis of persons with late-onset AD from 13 public datasets (e.g., ADNI, ROADMAP, and ADC1–3) identified a statistically significant SNP-SNP interaction between rs762571 in the *PSAP* locus and rs2466176 in the *PEBP4* locus (OR = 1.25, *p* < 0.00001 after covariate adjustment), implicating PSAP in potential combinatorial genetic risk for AD [108].

Although the tertiary structure of PSAP has not yet been resolved through traditional structural biology techniques such as nuclear magnetic resonance spectroscopy, X-ray crystallography, or cryo-electron microscopy, we were able to visualize its predicted tertiary structure using AlphaFold [109]. In parallel, we employed AlphaMissense [110] to assess amino acid level mutational tolerance across the PSAP protein (Table S1). We found that missense variant pathogenicity is not evenly distributed across PSAP domains. A large proportion of likely benign and ambiguous variants were located outside the saposin domains (A–D), while likely pathogenic variants were more enriched within defined saposin domains. Notably, saposin D contained the highest percentage of likely pathogenic variants among all domains. In contrast, saposin C, although functionally important, showed a lower relative burden of likely pathogenic substitutions (Fig. 3B). These findings suggest domain-specific tolerance to missense variation, with saposin D being the most mutation-intolerant.

## PSAP: dual roles in CNS homeostasis

PSAP and saposins play a paramount role in CNS lipid metabolism and lysosomal network homeostasis. PSAP and saposins enable the breakdown and turnover of various bioactive sphingolipid species, including sphingomyelins, ceramides, and gangliosides. This is particularly important given that lipids constitute the majority of the brain’s dry weight (~ 50%) [111, 112]. Among these, sphingolipids are particularly enriched and contribute to myelination, neurite extension, and synaptogenesis. PSAP expression is abundant throughout the brain [113] and is high in neurons and glial cells [16]. PSAP exerts its primary functions through activation of its receptors G-protein coupled receptor 37 (GPR37) and GPR37L1, which are exclusively expressed in the CNS. These receptors regulate CNS maturation, maintain homeostasis, and protect against neurotoxicity [33, 84, 114, 115]. Together, these findings underscore PSAP’s central role in the brain.

Saposins activate a wide range of enzymes involved in sphingolipid metabolism. Mutations in genes encoding these enzymes result in a range of lysosomal storage and neurological disorders (Fig. 1). *GBA1* mutations cause GD [116] and are linked to PD [117], while *GALC* mutations lead to Krabbe disease, also known as globoid cell leukodystrophy [118]. *ARSA* mutations result in metachromatic leukodystrophy [119], and *GLA* mutations cause Fabry disease [120]. *GLB1* mutations cause GM1 gangliosidosis [69] and Morquio disease [69, 121]. Mutations in *ASAH1* lead to Farber disease and spinal muscular atrophy with progressive myoclonic epilepsy [122], *SMPD1* mutations underlie Niemann-Pick disease and have been linked to PD [123, 124], and *NEU1* mutations lead to sialidosis [125]. These mutations have a commonality: lysosomal dysfunction and lipid accumulation within organs including the brain, albeit varying in degree.

### Neuroprotective roles of PSAP

PSAP and saposin deficiencies frequently result in disorders that mirror the pathologies and symptoms of the aforementioned enzyme-related conditions such as lysosomal dysfunction, lipid accumulation, and neurodegeneration [86, 126–128]. Global Psap knockout mice such as those that can be obtained from Jackson Laboratory (*Psap*^tm1Suz^) results in neonatal lethality and impaired CNS development, leading to severe demyelination and neurodegeneration [129, 130]. CRISPR-mediated knockdown of PSAP in iPSC-derived neurons induces glycosphingolipid buildup in lysosomes, leading to the formation of iron-rich lipofuscin granules, a marker of aging [131]. These granules accumulate ferrous iron that promotes Fenton chemistry, resulting in elevated reactive oxygen species and lipid peroxidation. The oxidative stress culminates in ferroptotic cell death, a process not observed in undifferentiated iPSCs, neural progenitors, astrocytes, microglia, or HEK293 cells following PSAP loss [132].

Accumulation of amyloidogenic protein oligomers and aggregates such as Aβ, tau, and α-synuclein leading to neurodegeneration can be largely attributed to lysosomal clearance deficits [133]. Given PSAP’s central role in lysosomal function and trafficking, it is plausible that PSAP dysfunction may directly impair lysosomal homeostasis and lead to the accumulation of amyloidogenic proteins. Indeed, disrupted lysosomal trafficking of PSAP has been identified as a pathogenic feature in α-synucleinopathies such as PD [134] and in tauopathies such as AD [17] and frontotemporal lobar degeneration (FTLD) [135].

Inducible neuronal DA-specific, but not serotonergic-specific, *Psap* knockout in vivo leads to α-synuclein buildup, synaptic dysfunction, and neurocognitive behavioral deficits, alongside a marked reduction in key sphingolipid species such as sphingomyelins, ceramide-1-phosphate, and gangliosides as revealed by spatial lipidomics [18]. This α-synuclein buildup may stem from the loss of saposin C, which has been shown to regulate α-synuclein homeostasis by dislodging it from glucosylceramide-rich lipid membranes under lysosomal conditions [55]. Given that saposin C acts as the obligate lipid‐binding co-activator of GBA1, and *GBA1* variants exist in some persons with α-synucleinopathies and may cause disease manifestation [136, 137], the connection between GD and α-synucleinopathy seems plausible. Some people with GD develop parkinsonism with α-synuclein-positive brain inclusions [138], suggesting that PSAP dysfunction may contribute to overlapping lysosomal and α-synuclein-driven pathology. A potential mechanism between saposin C dysfunction and α-synuclein accumulation could stem from impaired cathepsin B-mediated cleavage of PSAP into saposin C. One study found that cathepsin B is responsible for this cleavage and that this process was disrupted in a PD mouse model. This likely results in reduced saposin C levels, decreased GCase activity, and subsequent α-synuclein accumulation [139]. Consistent with this, ambroxol, a small molecule chaperone shown to enhance GCase activity, was found to significantly increase saposin C and cathepsin D activity and restore lysosomal function in fibroblasts from persons with PD with *GBA1* variants [134]. These findings support a model where PSAP dysfunction and impaired saposin C production disrupt GBA1 enzymatic activity, ultimately promoting lysosomal failure and α-synuclein aggregation, as seen in both GD and PD.

PSAP and its derivatives accumulate in dystrophic neurites surrounding Aβ in multiple AD mouse models (APP^NL-G-F^, 5xFAD, APP/PS1ΔE9) and in human AD brains, where they co-localize with LAMP1-positive but cathepsin B- and D-deficient lysosomes. Because cathepsin D, and to a lesser extent cathepsin B [139], cleave PSAP to active saposins, this suggests that impaired hydrolase activity may hinder saposin maturation, thereby contributing to lysosomal dysfunction in AD pathology. Interestingly, the early accumulation of PSAP and its derivatives in lysosomes within dystrophic neurites occurred most prominently in AD brains at an earlier neuropathological stage (Braak stages I and II) [17]. This is further supported by findings suggesting that PSAP alone, or as part of a PSAP–PGRN complex, co-localizes with Aβ in the brains of individuals with and without AD, with both positively correlating with brain Aβ plaque levels [16]. Additionally, CRISPR-mediated knockdown of *PSAP* in iPSC-derived neurons leads to accumulation of tau oligomers [140], the species preceding aggregates. These findings suggest that lysosomal dysfunction involving PSAP may be an early event in AD pathogenesis.

Interestingly, PSAP’s role in AD appears to be primarily related to Aβ, as PSAP levels were found to be lower in neurons containing neurofibrillary tangles (NFTs) from individuals with AD and FTLD compared to neurons without NFTs. PSAP levels also negatively correlated with the area occupied by phosphorylated tau at Threonine 231 (pT231-tau) [15]. In both PRGN-deficient mice and human FTLD postmortem brain tissue, PSAP fails to reach lysosomes, leading to elevated circulating PSAP levels, lysosomal dysfunction, and neuronal degeneration [135]. This suggests that PRGN may be necessary to regulate PSAP trafficking and homeostasis and that excess extracellular PSAP resulting from lysosomal dysfunction could contribute to neurotoxicity or serve as a compensatory mechanism to mitigate cellular stress.

### Neuropathological roles of PSAP

**Fig. 4:**
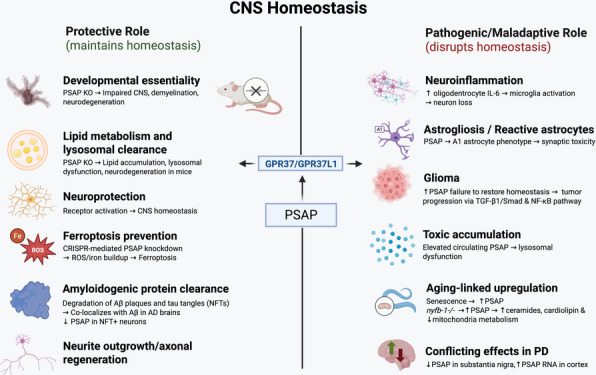
Dual roles of prosaposin (PSAP) in the central nervous system (CNS) homeostasis: protective versus maladaptive outcomes. This schematic illustrates the opposing roles of PSAP in CNS physiology. The central axis denotes PSAP-GPR37/GPR37L1 receptor signaling as a critical regulatory hub. Left panel—protective role (green): PSAP maintains homeostasis through multiple mechanisms, including lipid metabolism and lysosomal clearance (supported by PSAP knockout studies in mice), neuroprotection via GPR37/GPR37L1 receptor activation, and clearance of pathological proteins such as α-synuclein, tau, and Aβ. PSAP also promotes neurite outgrowth and axonal regeneration (e.g., TX14(A)-induced ERK signaling), prevents ferroptosis (via suppression of ROS and iron buildup), and is essential for CNS development and myelination. Right panel—pathogenic/maladaptive role (red): Elevated or dysregulated PSAP, when it is unable to function, may disrupt homeostasis, contributing to neuroinflammation (e.g., IL-6-driven microglial activation), glioma progression (via TGF-β1/Smad and NF-κB pathways), lysosomal dysfunction from toxic accumulation, and A1 astrocyte-driven synaptic toxicity. PSAP expression may be related to aging, and may exhibit region-specific and conflicting effects in Parkinson’s disease. Abbreviations: Aβ, amyloid beta; CRISPR, clustered regularly interspaced short palindromic repeats; ERK, extracellular signal-regulated kinase; GPR37, G protein-coupled receptor 37; GPR37L1, G protein-coupled receptor 37-like 1; KO, knockout; NFYB-1, nuclear transcription factor Y beta subunit 1; NF-κB, nuclear factor kappa-light-chain-enhancer of activated B cells; PD, Parkinson’s disease; ROS, reactive oxygen species, TGF-β1, transforming growth factor beta 1. Created with BioRender.com

While PSAP has been widely studied for its neuroprotective roles in maintaining brain homeostasis, it is important to note that PSAP’s function may be context-dependent (Fig. 4).

In mesenchymal glioblastoma, PSAP is highly expressed and secreted, promoting tumor invasion and epithelial-mesenchymal transition through the TGF-β1/Smad signaling pathway [141]. In primary glioma, PSAP is highly expressed and secreted in clinical glioma specimens, glioma stem cells, and glioma cell lines, where it promotes tumorigenesis through activation of the TLR4/NF-κB signaling pathway [142]. In both studies, higher PSAP expression predicted poorer prognosis. Notably, a recent study showed that PSAP acts on neural stem cells to maintain them in deep quiescence [143]. Higher PSAP levels in brain cancer may reflect a compensatory response: dysfunctional PSAP signaling (e.g., impaired receptor binding or downstream activity) or damage may cause cells to upregulate PSAP expression in an attempt to restore homeostasis. Failure to do so may lead to detrimental effects. This is partially supported by findings showing that PSAP’s receptor GPR37 is highly expressed in gliomas [144]. However, this remains largely speculative until further studies are conducted. In contrast, in non-brain cancers such as prostate and breast cancer, PSAP has demonstrated conflicting roles, acting as either a tumor promoter or suppressor depending on the cellular context and signaling environment [145].

Inositol polyphosphate-5-phosphatase D (INPP5D) is a phosphatase predominantly expressed in microglia, where it negatively regulates PI3K–AKT signaling pathways downstream of immune receptors. GWAS have identified variants in *INPP5D* associated with AD risk, yet functional studies have yielded conflicting results. In some models, *Inpp5d* deletion reduced amyloid plaque burden and enhanced microglial clearance, while in others, its loss exacerbated pathology. Recent single-cell transcriptomic analyses have shown that complete loss of *Inpp5d* disrupts microglial homeostasis, alters intercellular signaling, and drives widespread transcriptional changes including the upregulation of PSAP [146]. These findings suggest that PSAP may be involved in neuroinflammation including microglial and astroglial activation.

Indeed, some studies suggest that PSAP-related signaling in glial cells may lead to neuroinflammation and neurotoxicity, especially under normal conditions. Transcriptomic analysis of the hippocampal-entorhinal system in AD postmortem brains revealed significant upregulation of PSAP signaling in entorhinal cortex neurons. This PSAP, when administered to primary rat astrocytes, promoted astrogliosis and induced an A1-like reactive astrocyte phenotype. When co-cultured with primary rat neurons, these reactive astrocytes exhibited neurotoxic effects and impaired synaptic function on neurons [147]. Complementing these findings, PSAP binding to GPR37 on oligodendrocytes was shown to provoke a sharp rise in interleukin-6 (IL-6), setting off a feed-forward inflammatory loop in which oligodendrocyte-derived IL-6 activates microglia, amplifies neuroinflammation, and drives loss of DA neurons. Selective deletion of *Gpr37* in primary oligodendrocytes or knock-down of *Il-6* blocks this cascade, preserves nigral neurons, and prevents motor and pain deficits, indicating that the PSAP-GPR37-IL-6 axis is both necessary and sufficient for the inflammatory component of degeneration. Parallel human single-cell RNA-seq analyses revealed elevated GPR37 expression in oligodendrocytes of the substantia nigra in both PD and control individuals, with significantly higher expression in PD [30, 147]. These findings suggest that neuron-derived PSAP may act in a paracrine fashion on GPR37 receptors located on astrocytes and microglia, exacerbating neuronal loss and contributing to disease progression potentially through IL-6.

Of note here is that conditions involving neural trauma or damage such as CNS injury are known drivers of local and systemic inflammation [148]. These conditions have been shown to consistently increase *Psap* gene expression in areas of injury including spinal and facial nerves and the brain [21, 149–152]. The subacute phase of traumatic brain injury leads to PSAP upregulation, particularly in astrocytes activated by microglia. Functional assays show that PSAP specifically promoted neurite outgrowth in iPSC-derived neurons [153]. In support of this, a seminal study [154] demonstrated that dorsal root ganglion macrophages promote axonal regeneration following nerve injury via PSAP-mediated signaling through GPR37/GPR37L1 receptors. Single-cell RNA sequencing revealed that macrophage-derived PSAP likely mediates intercellular communication with satellite glial cells through GPR37L1, suggesting a potential glioprotective mechanism. To validate macrophage-satellite glial cell signaling, dorsal root ganglion explants from macrophage-depleted mice were treated with prosaptide TX14(A), a PSAP-derived peptide that binds GPR37 and GPR37L1. TX14(A) induced ERK phosphorylation in satellite glial cells and enhanced neurite outgrowth, consistent with established beneficial roles of PSAP and prosaptides in nerve regeneration (see below). We believe that these observations suggest context-dependent roles for PSAP, wherein increased signaling and secretion under acute CNS injury or regenerative conditions may confer beneficial effects, whereas its sustained or inappropriate activation in the absence of a need for regeneration might instead drive pathological neuroinflammation and/or neurotoxicity.

Additionally, in direct contrast to the results (described above) [18] showing that *Psap* depletion in DA-neurons leads to lysosomal dysfunction, α-synuclein accumulation, and neurodegeneration, another study [30] reported that *Psap* depletion in the brain led to the complete opposite effects. Both studies also reported conflicting and region-specific findings on PSAP gene expression and protein levels in brains of persons with PD. The former [18] reported that PSAP expression (using immunohistochemistry) is decreased in the postmortem substantia nigra of persons with PD, while the latter [30] reported increased *PSAP* gene expression in the brain of persons with PD. It is unclear why the two studies report completely conflicting findings, and further investigations are warranted.

Notably, PSAP may also have a detrimental role during aging. One study reported that loss of nuclear transcription factor Y, beta subunit 1 (NFYB-1) in *Caenorhabditis elegans* (*C. elegans*) profoundly reduces mitochondrial metabolism and lifespan. *nyfb-1* deficiency was found to upregulate PSAP’s ortholog, *spp-8*, leading to higher ceramide and cardiolipin levels. Remarkably, RNAi against *spp-8* or dietary cardiolipin supplementation restores respiration, mitochondrial morphology, and longevity in *nyfb-1*-deficient *C. elegans*, suggesting that SPP-8 overexpression is harmful [155]. Why this occurs in *C. elegans* remains unclear. During aging, *Psap* expression has been shown to be increased in hearts of aged (24 months) compared to young rats (6 months). Analysis of senescent human umbilical vein endothelial cells (HUVECs) and human dermal fibroblasts, defined by a population doubling greater than 52, revealed elevated *PSAP* gene expression. Additionally, inducing senescence in HUVECs using hydrogen peroxide or interferon-γ also increased PSAP protein levels [156]. Future studies are warranted to confirm these findings.

## PSAP as potential biofluid biomarker

**Fig. 5:**
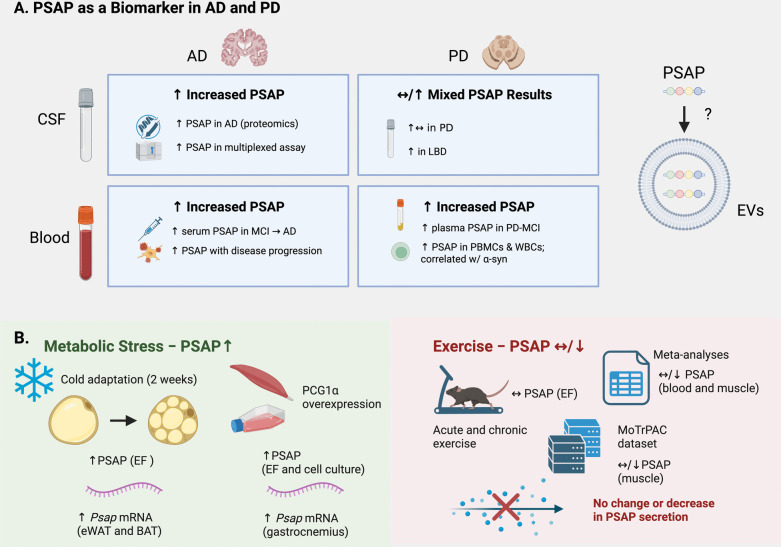
Prosaposin (PSAP) as a biomarker in neurodegenerative disease and its regulation by metabolic stress and exercise. **A** Summary of human studies measuring PSAP in Alzheimer’s disease (AD) and Parkinson’s disease (PD). In AD, PSAP is consistently increased in cerebrospinal fluid (CSF) and blood, as detected by proteomics and multiplex immunoassays. Serum PSAP increases from mild cognitive impairment (MCI) to AD and correlates with disease progression. In PD, CSF findings are mixed, but increased PSAP levels are observed in Lewy body dementia (LBD). In blood, plasma PSAP is elevated in PD-MCI and peripheral blood mononuclear cells (PBMCs) and white blood cells (WBCs), correlating with α-synuclein levels. The potential association of PSAP with extracellular vesicles (EVs) remains unclear. **B** PSAP expression increases in response to metabolic stress but is reduced or unchanged with exercise. Cold adaptation for 2 weeks elevates PSAP protein levels in inguinal fat extracellular fluid (EF) vs. epididymal white adipose tissue (eWAT) EF and increases *Psap* in iWAT and brown adipose tissue (BAT). Overexpression of PGC1α increases PSAP levels in gastrocnemius EF and cell culture medium of myotubes. In contrast, both acute and chronic exercise result in no change or a decrease in PSAP expression in blood and skeletal muscle, as shown by multiple meta-analyses (https://metamex.serve.scilifelab.se/app/metamex, https://www.extrameta.org/) and the rat 6-month treadmill Molecular Transducers of Physical Activity Consortium (MoTrPAC) dataset (https://motrpac-data.org/gene-centric). Created with BioRender.com

PSAP has been identified as a biomarker in several cancers [145]. Its levels are modulated by stress, with reductions observed in both plasma [157] and the hippocampus [158], correlating with stress-related behaviors [157]. Conversely, elevated PSAP levels have been linked to adverse outcomes, such as in sepsis, where increased blood PSAP levels predicts organ dysfunction and failure [159]. PSAP levels were higher in urinary EVs of individuals with coronary artery disease [160] and in urinary samples from patients with neurogenic bladder [161]. Additionally, decreased PSAP levels have been found in the cingulate cortex of persons with schizophrenia [162], dystrophic mouse brain [163], and tears of persons with multiple sclerosis [164]. While PSAP is relatively well studied in various systemic conditions, studies on its levels in the biofluids of individuals with neurodegenerative diseases are scarce (Fig. 5A).

In PD, PSAP levels appear to be higher in plasma [18], where significance was only reached in persons with PD and mild cognitive impairment (PD-MCI), but not in CSF or in PRGN or PSAP-PRGN complexes [18]. Notably, PSAP levels did not correlate with neurofilament light chain (NfL), a widely used marker of neuroaxonal injury [165]. Another study used a multiplexed proteomic assay and reported significantly higher CSF PSAP levels in persons with Lewy body dementia, but not PD [31], suggesting a connection between cognitive decline and PSAP levels. However, another study reported increased CSF PSAP levels in persons with PD [30]. One study reported that PSAP, saposin C, and α-synuclein levels were higher in white blood cells from persons with PD, with PSAP and saposin C significantly correlating with α-synuclein levels [166], while another reported that PSAP expression was higher in peripheral blood mononuclear cells of individuals with PD [167].

In AD, three studies suggest that PSAP levels are higher in the CSF and plasma, with levels increasing from the preclinical to dementia stages, indicating a potential correlation with disease progression. The first CSF proteomics study reported significantly higher PSAP levels in both preclinical and clinical AD compared to MCI and controls [168], while the second CSF study performed a multiplexed protein assay and reported significantly higher levels in AD vs. controls [31]. A serum proteomics study reported a 200% increase in PSAP levels following phenoconversion from MCI to AD [32]. PSAP’s receptor, GPR37, has also been identified by proteomics to be higher in the CSF of the *Shr72* rat model of tauopathy [169].

Given PSAP’s well-established role in neurodegenerative disease, both as a driver of pathology through dysfunction and as a gene whose mutation leads to several neurodegenerative disorders, there is a strong rationale for exploring it as a diagnostic and/or prognostic biomarker, especially when coupled with GPR37 concentrations, as they have shown promise for α-synucleinopathies [170]. We recommend that future studies conduct more targeted analyses of PSAP in the biofluids of individuals with neurodegenerative diseases, with particular emphasis on EVs. EVs are increasingly recognized for their ability to encapsulate and preserve cell-state-specific signals within a protective phospholipid bilayer, shielding their molecular cargo from enzymatic degradation. They are growing as biomarker tools for neurodegenerative disorders including AD [171], PD [172, 173], and ALS [174, 175]. Additionally, a multi-omic framework integrating genomics, transcriptomics, and proteomics would allow a more complete characterization of PSAP regulation and function [176].

## PSAP responds to metabolic stress but is not an exerkine, exercise-responsive molecule, nor a molecular transducer of exercise

Physical activity and exercise offer broad neuroprotective benefits, including reduced risk and improved prognosis for several neurodegenerative disorders such as AD and PD [177, 178]. These effects may be partly attributed to exercise-induced bioactive factors, or “exerkines,” that may mediate peripheral-to-brain communication with a few candidates such as cathepsin B (CTSB) [179], irisin (FNDC5) [180], clusterin [181], and platelet factor 4 (PF4) [182] showing some evidence for brain-targeting effects. Both full-length PSAP and saposin C are present at high levels in plasma and serum and at smaller levels in breast milk, semen, and pancreatic juices [86]. Moreover, PSAP secretion is enhanced under conditions of cellular stress, including sciatic and spinal nerve transection [27]. But to date, no study has identified PSAP as an exerkine, nor as an exercise-responsive molecule or a molecular transducer of exercise adaptation.

While the origins of circulating PSAP remain poorly understood, recent evidence [28] suggests that the gastrocnemius and inguinal white adipose tissue (iWAT) may contribute to its release into the extracellular fluid (EF) under conditions of metabolic stress (Fig. 5B). Proteomic analysis of gastrocnemius extracellular fluid (EF) from mice overexpressing PGC1α in skeletal muscle under the muscle creatine kinase (MCK) promoter, as well as thermogenic iWAT EF from wild-type mice exposed to a 2-week cold adaptation treatment, revealed elevated PSAP levels compared to wildtype controls and epididymal WAT (eWAT), respectively. The same study found that the former condition increased *Psap* gene expression in the gastrocnemius, whereas the latter increased its expression in iWAT and brown adipose tissue (BAT). Forced PGC1α expression in myotubes led to increased PSAP secretion into the cell culture medium, while forced PSAP expression in primary iWAT cells upregulated thermogenic genes (e.g., *UCP1*), oxidative phosphorylation genes, and enhanced both overall and basal respiration, suggesting that PSAP may modulate metabolic activity and contribute to thermogenic remodeling under stress conditions.

Although overexpression of PGC1α has been well-documented to induce an exercise-like state in skeletal muscle [183], and one could speculate that PSAP may be an exerkine or an exercise-responsive molecule based on the aforementioned results, exercise did not contribute to PSAP circulation levels. In the same study [28], proteomic analysis did not identify PSAP as a differentially altered protein in the gastrocnemius EF harvested from mice subjected to an acute (3-day) treadmill-running protocol relative to sedentary controls. Additionally, multiple large-scale meta-analyses (e.g., MetaMEx [184], ExtraMeta [185]) show that PSAP either decreases or remains unchanged with both acute and chronic exercise in skeletal muscle and blood and, in some cases, increases with inactivity in skeletal muscle.

In large-scale datasets such as the Molecular Transducers of Physical Activity Consortium (MoTrPAC), which performed multi-omics profiling across 20 tissues from 6-month-old rats subjected to 8 weeks of progressive treadmill training [186], exercise significantly decreased PSAP protein levels in WAT in males during the first 4 weeks before returning to baseline by week 8. Despite these changes in protein abundance, exercise did not alter *Psap* gene expression in WAT in either sex throughout the 8 weeks. In the vastus lateralis, *Psap* gene expression consistently decreased across all time points in both males and females, while it remained unchanged in the gastrocnemius [29]. Moreover, despite a plethora of studies performing multi-omics analyses on tissues and biofluids from acutely and chronically exercised humans and animal models, PSAP has not been highlighted as a significantly upregulated molecule at either the protein or gene expression level due to exercise. In fact, PSAP appears to decrease in response to exercise; a single acute bout of treadmill running in middle-aged adults reduced plasma PSAP levels, as demonstrated by proteomic analysis [187].

And while a study that overexpressed TFEB specifically in skeletal muscle of 5xFAD mice, aiming to mimic an exercise-like state, reported elevated PSAP levels via Western blotting (WB) in quadriceps and brain and ELISA in plasma [188], it is important to note several limitations. The quadriceps and brain WBs for PSAP were severely underpowered (*n* = 3 per group, no technical replicates), and the brain WB was faint. Although one study [189] reported PSAP immunoreactivity in multiple organs of female rats, including localization to skeletal muscle, the antibody used targeted a 22-amino-acid region within saposin C, raising specificity concerns. The study did not include data for several of the organs it claimed to examine, lacked quantitative analyses and omitted essential methodological details, limiting the reliability and interpretability of the findings. Importantly, 25 years have passed, and we are unaware of additional studies reporting similar findings.

Collectively, these findings clearly suggest that PSAP may respond to metabolic stress, but is not an exerkine, nor is it a skeletal muscle–specific exercise-responsive molecule or a molecular transducer of exercise adaptation. It is also unclear why adipocytes would release PSAP in response to cold adaptation, especially given that its known receptors, GPR37 and GPR37L1, are expressed exclusively in the CNS, raising questions about whether PSAP has any functional relevance in peripheral tissues under these conditions.

## PSAP and prosaptides as CNS therapeutics and peripheral-to-CNS delivery

Soon after their discovery, both PSAP and saposin C, but not other saposins, were found to promote neurite outgrowth *in vitro*^*90*^ sparking interest in their neurotrophic effects in vivo*.* Following this, the rat neurotrophic prosaptide sequence of saposin C was mapped to 12 amino acids at its N-terminal end (**LIDNNKTEKEIL**) [91].

**Fig. 6:**
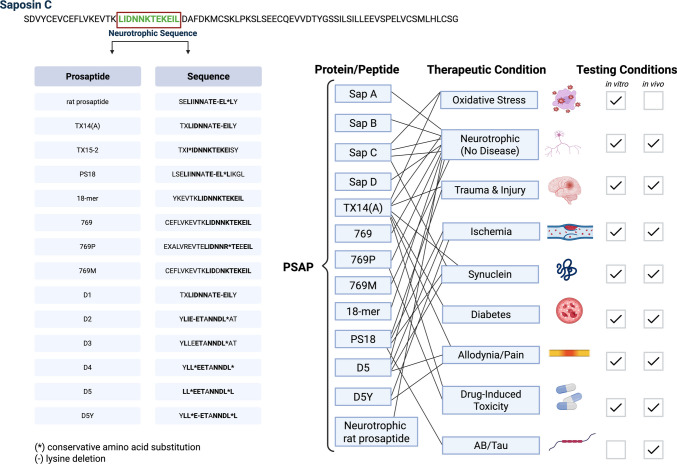
Overview of prosaptide peptides derived from the saposin C domain of prosaposin (PSAP) and their tested therapeutic applications. The full saposin C sequence is shown at the top, with the neurotrophic region (**LIDNNKTEKEIL**) highlighted in green and underlined. Left panel: Sequences of various bioengineered or truncated prosaptide peptides tested in previous studies. Conservative amino acid substitutions (*) and lysine deletions (-) are indicated. Bioactivity is noted where tested. Right panel: Mapping of each prosaptide or full saposin (A–D) to specific disease-relevant therapeutic conditions, including oxidative stress, trauma/injury, ischemia, synucleinopathies, diabetes, allodynia, drug-induced toxicity, and Alzheimer’s disease-related Aβ/tau pathology. Checkboxes indicate whether the peptide has been evaluated in vitro, in vivo, or both. Peptides derived from the neurotrophic region, such as TX14(A), 769, and PS18, show diverse functional relevance across multiple disease models. Created with BioRender.com

The neurotrophic rat prosaptide (SE**LI**I**NN**ATE-EL^*^LY, - denotes deletion of the lysine and * denotes a conservative substitution) was not tested extensively as early experiments showed that it has fewer in vitro and in vivo neurotrophic effects in comparison to TX14(A) [190]. Though several prosaptides have been studied, the most studied prosaptides are the 14-mer prosaptide TX14(A) (TX**LIDNN**A**TE-EIL**Y) > the 22-mer prosaptide 769 (CEFLVKEVTK**LIDNNKTEKEIL**) and 769P (EXALVREVTELIDNNR^*^TEE**EIL**) > the 18-mer peptide PS18 (LSE**LI**I**NN**ATE-EL^*^LIKGL) > retro-inverso peptide fragment D5 (LL^*^E-ETANNDL^*^L). PSAP, saposin C, and prosaptides have been shown to exert strong neuroprotective and neurotrophic effects in several in vitro and in vivo (except 22-mer) using male–female young–aged models of oxidative stress, CNS injury, ischemia, AD, PD, diabetes, allodynia and drug-induced neurotoxicity **(**Fig. 6**)**. Most of these effects have been recognized for over two decades, and some are reviewed elsewhere [86]. Rather than reiterate well-established findings, we provide a very detailed summary of these studies in Table 2 and shift our focus to evaluating whether exogenous prosaptides can cross the blood–brain barrier (BBB) following peripheral delivery to enable CNS penetration for therapeutic development.

**Table 2:** Summary of studies investigating prosaposin (PSAP) prosaptides as therapeutic agents across disease models ande experimental contexts

PMID	Study	Year	Country	Group	Model	Protein/peptide	Administration	Dose	Purification	Effect
No disease										
7937812	O’Brien et al.	1994	USA	In vitro: NS20Y, SK-N-MC and PC12M cells	None	Human milk PSAP; recombinant PSAP; saposins A–D	Addition to cell culture directly	Neurite growth assay: dose response of 0–0.6 ug/mL, or treatment with 100 ng/mL; ChAT activity assay: 200 ng/mL Binding studies: 0–2.5 nM 125I-labeled Sap C	Recombinant PSAP prepared by insect cell/baculovirus expression, Sap A, B, C, D, and milk PSAP were also prepared as previously described (Hiraiwa et al. 1993, 8323276; Morimoto et al. 1990, 2127157)	1) In the dose–response experiment, recombinant human PASP, Sap C, and purified human milk PSAP, but not Sap A, stimulated neurite-growth in NS20Y but not PC12M cells 2) 100 ng/mL Sap C, but not Sap A, increased proportion of NS20Y neurite-bearing cells 3) Sap C and PSAP, but not Sap A, increased ChAT activity in SK-N-MC cells 4) Binding assay demonstrated that Sap C binds to two putative receptors on NS20Y cells with high (Kd = 19 pM) and low affinity (Kd = 1 uM) 5) PSAP and Sap C, but not A, B or D, increased protein tyrosine and threonine protein phosphorylation
7768361	O’Brien et al.	1995	USA	In vitro: NS20Y and SK-N-MC cells in vitro: Primary granule cells from P8 rat cerebella (Thangnipon & Balázs 1992, PMID: 1538824)	None	PSAP; Saposin A; Saposin C; Saposin D; 18-mer (YKEVTKLIDNNKTEKEIL) prosaptide; 769	Addition to cell culture directly	100 ng/mL for for cell viability and ChAT activity 0 to 10 ng/mL for the neurite outgrowth assay	Sap C peptides synthesized as per Weiler et al. 1993 (PMID: 8292489); other peptides synthesized by solid-phase synthesis and shorter peptides from Genosys Biotechnologies. Peptides were purified by HPLC on Vydac C4 column to > 95% purity	1) 100 ng/mL PSAP, Sap C, 18-mer prosaptide and 769, but not Sap D or 769 M, reduced cell death in NS20Y cells 2) 100 ng/mL PSAP, Sap C and 769, but not Sap A, increase choline acetyltransferase activity in SK-N-MC cells 3) 100 ng/mL PSAP, Sap C or 769, increase NS20Y cells neurite-growth 4) Sap C, 18-mer prosaptide and 769 bound to NS20Y cell surface with high affinity 5) 769 increased tyrosine and threonine protein phosphorylation in NS20Y cells 6) 18-mer prosaptide’s binding affinity to NS20Y cells was Kd = 70 pM and 6600 sites per cell, and saposin C’s binding affinity to NS20Y cells was Kd = 19 pM and 2000 sites per cell
9114068	Hiraiwa et al.	1997	USA	In vitro: primary Schwann cells from P1 Sprague–Dawley rats’ sciatic nerves, iSC cells (spontaneously transformed line from rat primary Schwann cells (iSCs)) (sex NA); CG4 oligodendrocytes	None	PSAP; Saposin C; TX14(A); 769P; 769 M	Addition to cell culture directly	PSAP (250 ng/mL); Sap A-D (50 ng/mL); TX14 (1, 5, 10, 20, 25, 50 or 80 ng/mL); 769 and 769 M (concentrations NA)	PSAP from Sf9 cells infected genetically modified to express human PSAP see (see Hiraiwa et al. 1993, PMID: 8323276); Sap C was isolated from the spleen of a person with Gaucher’s disease (see Morimoto et al. 1990, PMID: 2127157)	1) Saposins A, B, and D and 769 M are inactive peptides and did not enhance sulfatide content in primary Schwann cells 2) PSAP, saposin C, TX14(A), and 769P (concentration NA) increase sulfatide content in primary Schwann cells 3) 2.5 to 20 ng/mL TX14(A) increases sulfatide content in CG4 cells, and 10 ng/mL stimulated sulfatide content in CG4 cells the greatest 4) 1 or 5 nM TX14(A) increased MAPK p42 and p44 phosphorylation in primary Schwann and iSC cells 5) 25 and 50 nM TX14 did not increase primary Schwann cells’ proliferation 6) 1 and 5 nM TX14(A) prevented iSC Schwann cells' cell death
8737244	Misasi et al.	1996	USA	In vitro: NS20Y cells	None	PSAP; 769; 769 M	Addition to cell culture directly	100 ng/mL each	PSAP prepared from human milk, synthetic peptides commercially obtained purified by HPLC on Vydac C-4 column to > 95% purity	1) PSAP did not alter ganglioside pattern in NS20Y but it increased content of resorcinol positive bands 2) 769, but not 769 M, caused modification of ganglioside content but the other synthetic peptides did not
8780053	Kotani et al.	1996	Japan	in vitro: primary neurons from E17 male/female Wistar rats (see Hiraiwa et al. 1988, PMID: 3,359,332)	None	PSAP; Saposins A-D; PS18	Addition to cell culture directly	PSAP (200 ng/mL), SAP C (400 ng/mL) and PS18 (160 ng/mL)	Affinity chromatography from human milk using a monoclonal antibody for PSAP. Saposin A-D from bovine spleen using a similar method. PS18 was obtained from Sawady Technology (details NA)	PSAP, saposin C, and PS18 increased MAP2 + neuronal numbers and neurites
8954961	Campana et al.	1996	USA	In vitro: PC12 cells	None	PSAP; 769; 769 M	Addition to cell culture directly	Phosphorylation: 0, 0.001, 0.01, 0.1, or 1 nM PSAP, or 1 nM prosaptide; Time course: 0.1 nM PSAP or prosaptide	PSAP purified from human milk, 22-mer prosaptides were obtained from Anaspec with 97% purity by HPLC analysis	1) PSAP and 769 bind to PC12 cells 2) PSAP increases tyrosine phosphorylation proteins of approximately 200, 190, 100, 95, 55 and 45 kDa (only 0.1 nM tested) and induces MAPK p42 and p44 phosphorylation (all concentrations). This effect is reduced at the higher 1 nM PSAP dose 3) 769 increases MAPK p42 and p44 phosphorylation, but a ten fold higher molar dose of the prosaptide was needed for this effect compared to PSAP 3) MAPK phosphorylation peaked at 5 min and returned to baseline levels by 30 and 60 min for both PSAP and 769
9506474	Campana et al.	1998	USA	In vitro: iSC cells from rat primary Schwann cells (see Bolin et al. 1992, PMID: 1280693), primary Schwann cells from neonatal rats (see Assouline et al. 1983, PMID: 6605783) (age and sex NA)	None	TX14(A)	Addition to cell culture directly	10 nM	Synthesized to 98% purity by AnaSpec	1) TX14(A) increased ERK1 and ERK2 phosphorylation in primary Schwann cells, and the magnitude increase over control in p-ERK1/total ERK1 (18-fold) was greater than p-ERK2/total ERK-2 (threefold), and this effect was blocked by pertussis toxin 2) The GTP-binding protein inhibitor, GDP-bS blocked TX(14)A-induced ERK phosphorylation in iSC cells, indicating that TX14(A) acts through G-proteins 3) TX14(A)-induced ERK phosphorylation increased ERK catalytic activity in iSC and primary Schwann cells 4) TX14(A) increased tyrosine phosphorylation of the Shc isoforms p46, p52, and p66 in iSC cells, and it also increased PI(3)K in Shc immunoprecipitates compared to controls in iSC cells 5) The PI(3)K inhibitor wortmannin prevented TX14(A)-induced ERK phosphorylation in iSC cells 6) TX14(A) increased sulfatide levels 2.5-fold in primary Schwann cells compared to controls and this effect was blocked by pertussis toxin and reversible MEK inhibitor PD098059 7) TX14(A) increased ERK phosphorylation in primary Schwann cells, though to a lesser degree than in iSC cells, and this effect was blocked by PD098059. ERK1 and ERK2 phosphorylation was rapidly elevated by 5 min and returned to baseline by 30 min
11442168	Campana et al.	2000	USA	In vitro: adult female primary sensory neurons from Wistar rats lumbar, thoracic and cervical dorsal root ganglions (age NA) in vivo: motor endplates in male CD-1 mice (age NA)	None	TX14(A); neurotrophic rat prosaptide; D5	In vitro: addition to cell culture directly in vivo: injected on surface of right superior gluteus muscle 1 cm lateral to spinal column	in vitro: 0.5, 5, or 20 ng/mL TX14(A), or 0.5 ng/mL of neurotrophic rat prosaptide, TX14(A), or prosaptide D5 for 3 days in vivo: 20 ug of TX14(A), 769 or 769P for 7 days	TX(14), prosaptide 769 and prosaptide 769P were obtained from AnaSpec; The neurotrophic rat prosaptide and retro-inverso D5 were synthesized were synthesized on a PS3 Protein Technologies PeptideSynthesize and purified by reverse phase HPLC	1) 0.5, 5, and 20 ng/mL TX14(A) significantly increased proportion of neurite-bearing cells 2) 2) 0.5 and 5 ng/mL, but not 20 ng/mL, TX14(A) significantly increased growth index of neurites per neurite-bearing cell 3) 0.5 ng/mL TX14(A) and D5 increased neurite-bearing neurons and neurite length, but neurotrophic rat prosaptide only increased the percent of neurite-bearing neurons and did not impact neurite length 4) 769, 769P, and TX14(A) injections increased frequency of terminal endplate sprouting but did not increase sprouting at nodes of Ranvier
10991978	Taylor et al.	2000	USA	In vivo: male Sprague–Dawley rats (age NA); in vitro: NS20Y mouse neuroblastoma cells and SH-SY5Y human neuroblastoma cells	None	Wild-type prosaptide (TKLIDNNKTEKEIL), TX14(A), TX15-2, and Prosaptides D1, D2, D3, D4, and D5	In vitro: direct addition to cell culture for neurite outgrowth experiment or co-incubation with prepared cell membranes for [35 S]GTPγS binding assay; in vivo: for transport experiments, the right jugular vein and left carotid artery of the rats were exposed and 125I-Prosaptide D4 was injected along with the radiolabeled albumin (plasma marker)	In vivo stability and blood–brain barrier transport experiments: 10 million cpm of 125I-Prosaptide D4; [35 S]GTPγS binding assay: 10 ng/mL of prosaptides D1–D5 were co-incubated with cell membranes; neurite outgrowth assay in NS20Y: exact dose of wild-type prosaptide, TX14(A), TX15-2, and prosaptides D1-D5 were not given(?) but ED50 was determined	Obtained from AnaSpec at > 95% purity. Prosaptide D4 iodinated with iodobeads (Pierce) and Na 125I using 0.25 mCi/12.5 μg of peptide and 8 min labeling time, and radiopurity was confirmed by HPLC	1) Prosaptide D5 had the lowest ED50 (0.2 +—0.01 ng/mL) on NS20Y neurite outgrowth (four times more active than TX14(A)), while wild-type prosaptide had the highest ED50 (1.4 + to 0.11 ng/mL), Prosaptide D1 had no biological activity on neurite outgrowth. Overall, prosaptides D2, D3, D4, and D5 were biologically active with ED50 ranging from 0.2 to 0.8 nM 2) 10 ng/mL Prosaptides D2, D3, D4, and D5 stimulated [35 S]GTPγS binding to cell membranes while D1 did not, and the inactive L-amino acid prosaptide 14 M1 inhibited prosaptide D5’s effects on binding 3) Prosaptide D4 was present in serum or brain for the 60 min that were examined post-injection in vivo, and it had a half life of 3.3 min in serum, and unidirectional influx rate constant Ki of 2.5 × 10^−4^ ml/(g*min) for brain transport
10773009	Taylor et al.	2000	USA	In vitro: NS20Y mouse neuroblastoma cells and SH-SY5Y human neuroblastoma cells; in vivo: male Sprague–Dawley rats (age NA)	None	Wild-type prosaptide, 14M1, 14M2, 14 M, TX14(A), TX14(C), TX14(D), TX15-1, TX15-2, TX15-3, Prosaptide S, Prosaptide Q, 12 A, 11 A, 11, 10, and 9	In vitro: direct addition to cell culture for neurite outgrowth experiment or co-incubation with prepared cell membranes for [35 S]GTPγS binding assay; in vivo: for transport experiments, the right jugular vein and left carotid artery of the rats were exposed and 125I-peptide was injected along with the radiolabeled albumin (plasma marker). For in vivo stability experiments, 125I-peptide was injected with PBS	In vivo stability and blood–brain barrier transport experiments: 2 million cpm of 125I-Prosaptide D4; [35 S]GTPγS binding assay: 10 ng/mL of prosaptides were co-incubated with cell membranes; neurite outgrowth assay in NS20Y: performed as per O' Brien et al. 1995 (7768361) where 0–10 ng/mL of each prosaptide was added to cell culture in a dose–response experiment	Obtained from AnaSpec at > 95% purity. Prosaptide D4 iodinated with iodobeads (Pierce) and Na 125I using 0.25 mCi/12.5 μg of peptide and 8 min labeling time, and radiopurity was confirmed by HPLC	1) Wild-type prosaptide, 14M3, TX14(A), TX15-1, TX15-2, prosaptide S, prosaptide Q, 12 A, 11 A, and 11 stimulated neurite outgrowth of NS20Y cell cultures. 14M1, 14M2, TX14(C), TX14(D), TX15-3, 10, and 9 were not bioactive 2) 10 ng/mL TX14(A), TX15-1, TX15-2, prosaptide S, prosaptide Q, 12 A, and 11 A stimulated [35 S]GTPγS binding to cell membranes, while TX14A(C), TX14(D), 14M1, TX15-3, and 10 did not. Wild-type prosaptide, 14M2, 14M3, 9, and 11 were not tested 3) TX14(A) degraded to 65% of original after 2 min in vivo in serum, and at 60 min there was < 30% intact remaining in serum 4) TX14(A) degraded rapidly to < 30% by 10 min in the brain and was undetectable by 60 min 5) TX15-2 degraded to < 10% intact by 10 min in serum, but increased over time in the brain where there was 20% intact at 2 min but 50% intact at 60 min 6) Prosaptide S and Q were less table in the brain than TX15-2, but prosaptide S had slower serum degradation (90% intact at 2 min, 45% intact at 10 min) compared to TX15-2 and TX14(A) 7) TX14(A) serum clearance had a half life of 4.2+ to 0.3 min and TX15-2 serum clearance had a half life of 2.5+ to 0.3 min. TX14(A) had a Ki of 1.25 × 10^−3^ mL/(g*min) for blood-barrier crossing and TX15-2 had a Ki of 4.86×10^−3^ mL/(g*min) and adding excess unlabeled peptide into the injection did not significantly inhibit the Ki values
11360264	Rende et al.	2001	USA	In vitro: L6 myoblast cells in vivo: adult female Sprague–Dawley rats (age NA)	None	TX14(A)	In vitro: Addition to cell culture directly in vivo: Intraperitoneal injections	In vitro: 1, 5, 10, 50, or 200 ng/mL in vivo: 1 mg/kg thrice-weekly	The peptide was obtained from AnaSpec to 98% purity	1) TX14(A)-treated mice had reduced weight loss in the EDL muscle after sciatic nerve ligation but TX14(A) had no effect on soleus muscle weight in the injured limb, or soleus or EDL muscle weight in the uninjured limbs 2) 1 ng/mL TX14(A) increased rate of L6 myoblast fusion, 5 ng/mL TX14(A) only increased L6 myoblast fusion when given every third day, and higher concentrations (20–200 ng/mL) did not alter the final percentage of fusion 3) TX14(A), regardless of dose or administration schedule, did not alter cell L6 myoblast proliferation as measured by cell counting
11356264	Hiraiwa et al.	2001	USA	In vitro: immortalized (adult; see PMID: 1,280,693) and normal (neonates, see PMID: 6,605,783) primary Schwann cells from Sprague–Dawley rats sciatic nerve (age and sex NA) in vivo: Neonate Sprague–Dawley rats (age and sex NA)	None	Prosaptide D5	in vitro: Addition to cell culture directly; in vivo: Subcutaneous injection into rat neonates	In vitro: ERK phosphorylation in iSC cells: 5 nM PSAP, 5 nM SapC, 25 nM TX14, 1 nM D5; dose–response in immortalized Schwann Cells: 0, 12.5, 25, 50, 100, 200 nM TX14, or 0, 0.1, 0.5, 1, 2 nM D5 in vivo: 100 ng/g D5 every 48 h until 28 days of age	Peptides synthesized and purified as per Taylor et al. ( 200,	1) In vivo: D5 significantly increased sulfatide brain concentrations at days 7, 10 and 14, but not 21 and 28 2) In vivo: D5 significantly increased sulfatide concentrations in sciatic nerves only across all timepoints 3) In vitro: D5 increased ERK-1 and ERK-2 phosphorylation in immortalized Schwann cells. D5 also increased ERK phosphorylation in normal Schwann cells at doses as low as 100 pM. These effects were larger than PSAP, Sap C, and TX14(A)
23690594	Meyer et al.	2013	USA	In vitro: HEK293T cells, cultured primary cortical astrocytes from P1-2 mouse cortices (sex NA)	None	TX14(A)	Addition to cell culture directly	1) 1 uM TX14(A) for endocytosis experiment HEK293 GPR37 or GPR37L1 2) 100 nM TX14(A) for HEK293 GPR37 or GPR37L1 transfected cells for 10 min 3) 100 nM TX14(A) for primary astrocyte experiment:	Purchased from Anaspec (details NA)	1) TX14(A) binds GPR37 and GPR37L1 and causes their endocytosis in HEK293 cells as shown by confocal microscopy 2) 100 nM TX14(A) increased pERK in transiently transfected HEK293T with either GPR37 or GPR37L1 cells. This effect was modulated by a Gαi/o-dependent mechanism leading to decreased cAMP levels 3) TX14(A) increased Gαi/o coupling to GPR37 and GPR37L1 4) TX14(A) increased ERK phosphorylation in primary cortical astrocytes. This effect was abolished by GPR37 knockdown and joint knockdown of GPR37 and GPR37L1, but not knockdown of GPR37L1 alone 5) TX14(A) binds HEK293T GPR37 with EC50 = 7 nM and GPR37L1 with EC50 = 50 nM 6) GPR37 is more expressed in astrocytes than in the whole brain 7) GPR37L1 is more expressed in the whole brain than in astrocytes
30260505	Liu et al.	2018	UK	In vitro: primary neurons from E18 rat cortices, primary astrocytes from P2 rat cortices, cerebellum, and brainstem; acute primary astrocytes from P12 rat cortices, and HEK293 cells	None	TX14(A)	Addition to cell culture directly	100 nM TX14(A)	HPLC to a purity of ≥ 95% (Tocris, Cat # 5151)	1) TX14(A) binds to GPR37L1/GPR37 to activate its signaling and decrease cAMP levels in primary astrocytes and neurons, but not HEK293 cells, suggesting that it primarily acts on CNS cells 2) GPR37L1 is highly expressed in primary rat astrocytes while GPR37 is expressed at a lower level
39269584	Taha et al.	2024	USA	In vivo: C57/B6L male young (3 months) mice	None	PS18	In vivo: subcutaneous injection daily for 7 days	2.0 mg/kg PS18	PS18 obtained from Phoenix Pharmaceuticals (324–431)	PS18-injected animals showed modest but unpowered and independently unvalidated increases in BDNF, SNAP25, and PSD95 in cortical synapatosome fractions. Additionally, there were nonsignificant trends towards increases for SYP1, SYT1, and SAP97 in the cortical synaptosome fractions
39913287	Ma et al.	2025	China	In vitro: primary mouse oligodendrocytes from newborn cerebral cortices (age & sex NA) in vivo: adult male mice 8–12 weeks Gpr37 global knockout (gKO) or WT mice	None	TX14(A)	Addition to cell culture directly or injection in mice	Oligodendrocytes: 30 or 300 nM, mice: 0.5 uL of TX-14 (1 ug/uL) for 12 consecutive days, microglia: co-culturing with TX-14-treated oligodendrocytes	TX-14 obtained from QYAOBIO	1) TX14(A) increased IL-6 in a dose-dependent manner in WT vs. Gpr37 gKO primary oligodendrocyte culture. This effect was blocked by inhibiting Pertussis toxin and MEK using the U1026 inhibitor, suggesting the effect is dependent on the GPR37/GPR37/Gαi/MEK pathway 2) TX14(A) caused degeneration of substantia nigra pars compacta neurons, impairment of movement coordination and mechanical allodynia in WT mice in comparison to Gpr37gKO mice
Oxidative stress										
18706485	Ochiai et al.	2008	Japan	in vitro: PC-12 cells	H2O2 (100 µM)	PSAP; Saposin C; TX14(A)	Addition to cell culture directly	100 ng/mL for PSAP, Sap C, and TX14A	PSAP and Sap C purified “as previously described” but no citation; Reverse HPLC using C4 column for TX14(A) (described in detail in PMID 2001789)	1) PSAP, Sap C, and TX14(A) rescued PC-12 cell viability from 100 µM H2O2 2) PSAP rescued H2O2-induced apoptosis (caspase 3 activity and phosphorylation of JNK and p38) to normal levels 3) PSAP inhibits H2O2-induced Akt phosphorylation and stimulates Erk phosphorylation between 10 and 30 min of H2O2 administration, suggesting that its downstream signaling involves these pathways 4) PSAP cells from H2O2 damage by increasing protein levels of -JUN and ATF3
23690594	Meyer et al.	2013	USA	In vitro: primary astrocytes from P1-2 C57BL/6 mouse cortices	H2O2 (500 µM)	PSAP	Addition to cell culture directly	100 nM PSAP	PSAP enriched in and purified from the HEK293T cells	PSAP rescued cortical astrocyte cell viability from 500 µM H2O2 via GPR37/GPR37L1-mediated ERK phosphorylation
30260505	Liu et al.	2018	UK	in vitro: primary neurons from E18 rat cortices, primary astrocytes from P2 rat cortices, cerebellum, and brainstem; acute primary astrocytes from P12 rat cortices, and HEK293 cells	H2O2 (250 μM), staurosporine (200 nM) or rotenone (100 nM) for 5 h	TX14(A)	Addition to cell culture directly	0, 50 or 100 nM TX14(A)	HPLC to a purity of ≥ 95% (Tocris, Cat # 5151)	1) 100 nM TX14(A) rescued primary astrocytes from oxidative stress caused by H2O2, staurosporine, or rotenone, an effect dependent on GPR37/GPR37L1 signaling 2) 100 nM TX14(A), but not 50 nM, protected cultured neurons from oxidative stress 3) TX14(A)-mediated rescue of oxidative stress in neurons and astrocytes is dependent on GPR37/GPR37L1 signaling
Trauma and injury										
8780031	Kotani et al.	1996	Japan	In vivo: male guinea pigs (age NA)	Sciatic nerve transection	PSAP	Collagen-filled nerve guide with PSAP in between both ends of the sciatic nerve after transection	2, 10, 20, 40, or 200 ng/mL	Affinity chromatography from human milk using a monoclonal antibody	1) 20 ng/mL PSAP increased regenerative nerve area threefold compared to control, but 40 ng/mL PSAP had no effect and 200 ng/mL suppressed nerve regeneration, indicating a biphasic, dose-dependent response 2) 20 ng/mL PSAP increased ratio of regenerating nerve fiber number to preoperative nerve fiber number, but 40 ng/mL and 200 ng/mL reduced this ratio, indicating a biphasic, dose-dependent response 3) 20 ng/mL PSAP increased spinal motor neuron and DRG neuron area and reduced chromatolysis, but did not alter the neuron number in the anterior horn or DRG 4) 20 ng/mL PSAP protected anterior motor neurons in spinal cord and small sensory neurons in DRG from atrophy and chromatolysis 5) 20 ng/mL PSAP increased area of spinal ChAT-positive neurons
10454138	Otero et al.	1999	USA	In vivo: adult Sprague–Dawley rats (sex and age NA)	Sciatic nerve ligation	TX14(A)	Subcutaneous injection and behavioral tests were conducted at 3, 24, 48, and 72 h thereafter	10, 50, 100 or 200 ug/kg	The peptide was synthesized by AnaSpec and isolated using reverse phase HPLC, > 95% purity	TX14 improved thermal hyperalgesia at doses of 50 and 100 ug/kg, but not 10 ug/kg, revealing a dose-dependent effect
10400252	Hozumi et al.	1999	USA	In vivo: female Sprague–Dawley rats (age NA)	Brain stab wound	PSAP	Intracerebral into the stab wounds	240 ng/day total for 3 days post-injury (120 ug/uL per day in each of the two stab wounds)	NA	1) PSAP improved acquisition in the escape response for the water maze task compared to vehicle (PBS) control mice 2) PSAP improved performance in the transfer test for the water maze task compared to vehicle (PBS) control mice 3) PSAP treatment significantly shrunk the stab wound area compared to vehicle (PBS) control mice 4) PSAP suppressed neuroinflammation compared to vehicle (PBS) control mice
10383054	Hiraiwa et al.	1999	USA	In vitro: primary Schwann cells from female Sprague–Dawley rats’ sciatic nerves (sex NA)	Sciatic nerve transection	TX14(A)	Addition to cell culture directly	Myelin markers experiment: 10 nM TX14(A); Schwann cell morphology experiment: 10 nM TX14(A); cell proliferation experiment: 0, 25, or 50 nm TX14(A)	The peptide was synthesized by AnaSpec and isolated using reverse phase HPLC, > 95% purity	1) 24 and 48 h of treatment with 10 nM TX14(A) significantly increased sulfatide levels in primary Schwann cells 2) 24 h but not 48 h of 10 nM TX14(A) treatment significantly increased GalT enzyme activity 3) TX14(A) treatment significantly increased GalT and P0 mRNAs 4) TX14(A) treatment caused cells to change from multipolar and irregular morphology to a uniform spindle-shaped bipolar morphology 5) TX14(A) did not significantly increase cell proliferation
25993033	Nabeka et al.	2015	Japan	In vivo: 10-week-old male Wistar rats	Kainic acid induced brain injury	PS18	Subcutaneous injection	0.2 and 2 mg/kg once a day for 3 consecutive days	Synthesized by Operon	1) 0.2 and 2 mg/kg PS18 increased response latency in the passive avoidance test in KA-treated animals, but it did not affect performance in the inclined screen tests across any group 2) 0.2 and 2 mg/kg PS18 reduced the number of injured neurons in the hippocampal CA1, CA3, CA4, and dentate gyrus (DG) regions 3) PS18 increased the number of intact synapses in the hippocampal CA1 region 4) PS18 did not affect pyramidal neurons in layer V of the cerebral cortex
30260505	Liu et al.	2018	UK	In vitro: primary astrocytes from P2 rat cortices, cerebellum, and brainstem	Scratch wound assay alone or combined with H2O2 (250 μM, 1 h), staurosporine (100 nM, 2 h) or rotenone (50 nM, 2 h)	TX14(A)	Addition to cell culture directly	100 nM TX14(A)	HPLC to a purity of ≥ 95% (Tocris, Cat # 5151)	1) PSAP depletion in cell culture media significantly slowed scratch wound closure 2) TX14(A) rescued scratch wound closure in PSAP-depleted media, an effect dependent on cAMP signaling as a proxy for GPR37L1/GPR37 activation 3) PSAP-depletion and TX14(A) administration did not affect astrocyte cell number (DAPI staining) or number of newly dividing cells (BrdU staining), indicating the wound closure effects seen is due to astrocyte motility and not astrocyte division 4) PSAP- and TX14(A)-dependent of wound healing through astrocyte migration is dependent on GPR37/GPR37L1 and cAMP downstream signaling
37153447	Khan et al.	2023	Japan	In vivo: commercial chickens (embryo developmental stages 18–20)	Spina bifida aperta	PS18	intra-amniotic treatment	0.5 ug PS18 in 100 uL saline	Synthesized by Eurofins Genomics	1) PS18 improved the neural tube restoration in the spina bifida aperta chicken model (fluorescence imaging) 2) PS18 rescued FBP and CR immunoreactivity in the point of fusion area and central canal 3) PS18 corrected neuroinflammatory astrocyte (GFAP), oligodendrocyte (O4), and microglia (IBA1) activity in the spinal cord’s ependymal layer 4) PS18 reduced apoptosis (lower caspase 3, higher Bcl-2) in pro-regenerative cells in the spinal cord. PS18 was also protective against secondary damage during the developmental period 4) PS18 rescued the chicks from postnatal movement deficits (leg joint movement ability, standing, sitting, walking), encouraged healing of the spina bifida aperta lesion, improved sensorimotor responses, and improved bowel movement
36763532	Feng et al.	2023	USA	In vitro: primary L4 dorsal root ganglia from 8-week old C57BL/6 mice treated with TX14(A) for 2 or 6 h (ERK phosphorylation experiments) in vitro: primary L4 dorsal root ganglia from 8-week-old C57BL/6 mice treated with PLX73086 to deplete macrophages or vehicle for 14 days, followed by TX14 administration (or vehicle) for 48 h (neurite outgrowth experiments)	Sciatic nerve full crushing	TX14(A)	Directly added to the dorsal root ganglia explant cultures	10 nM TX14(A)	Obtained commercially (MedChemExpress, Cat # HY-P1342)	1) TX14(A) increased ERK phosphorylation in some satellite glial cells 2) TX14 upregulated neurite outgrowth
Ischemia										
7980569	Sano et al.	1994	Japan	In vivo: male 12-week old Mongolian gerbils	Ischemia (occlusion of common carotid arteries for 3 min)	PSAP	7-day infusion to the lateral ventricles	100 or 240 ng/day	Affinity chromatography from human milk using a monoclonal antibody	1) PSAP rescues CA neuronal synapses (electron microscopy) and numbers (cresyl violet staining) 2) PSAP rescues neurocognitive behavioral deficits (passive avoidance task)
8780053	Kotani et al.	1996	Japan	In vivo: adult male Mongolian gerbils (age NA)	Ischemia (occlusion of common carotid arteries for 3 min)	PS18	PS18 7-day infusion to the lateral ventricles	PS18 7 or 20 ng/day for ischemia	Chemically synthesized from Sawady Technology, details NA	1) PS18 rescues CA1 neuronal numbers (electron microscopy and microscopy using cresyl violet) 2) PS18 rescues neurocognitive behavioral deficits (passive avoidance task)
10078882	Igase et al.	1999	Japan	In vitro: primary cortical neurons from E17 Wistar rats cerebral cortices in vivo: 12–13-week-old stroke prone-spontaneously hypertensive (SP-SH) rats	Focal cerebral ischemia	PS18	In vitro: directly added to cell culture in vivo: continuously infused by minipump in left lateral ventricle following middle cerebral artery occlusion	In vitro: 0.2, 2, 20, 200 fg/mL, or 2, 20, 200 pg/mL, or 2, 20, 200 ng/mL. in vivo: 4, 20, or 60 ng/day PS18 for 28 days	Chemically synthesized from Sawady Technology, details NA	1) On 2nd or 4th trial days, 20 or 60 ng/day PS18 educed mean escape latency (morris water maze test) in ischemic rats compared to vehicle, but 4 ng/day reduced mean escape latency only on the 2nd day 2) 60 ng/day PS18 did not alter body or brain temperature in ischemic rats 3) 4 to 60 ng/day PS18 effectively prevented secondary thalamic degeneration in ischemic rats and increased the left-to-right ratio of cerebrocortical area and thalamic ratio 4) PS18 increased the number of viable ventro-posterior thalamic neurons 5) PS18 only at doses between 2 fg/mL and 2 pg/mL, but not higher doses (20 pg/mL to 200 ng/mL) prevented FeSO4-induced neuron death (measured by MAP2 western blotting) 6) PS18 at doses of 2 or 200 fg/mL, but not higher doses (20 pg/mL to 200 ng/mL) reduced FeSO4-induced lipid peroxidation (TBARS concentration)
10852246	Lu et al.	2000	USA	In vitro: NS20Y in vivo: male Sprague–Dawley rats (age NA)	Focal cerebral ischemia	D5 and D5Y	In vitro: directly adding to cell culture; in vivo: systemic injection	In vivo experiments: D5Y: 200 ug/kg [125 I]D5Y at 10, 20, 40, 60 and 90 min; D5: 300 ug/kg at 3 or 6 h post-MCA occlusion; in vitro: 0, 0.2, 0.4, 0.6, 0.8, or 1 pmol/mL D5	Synthesized and purified by reverse phase HPLC	1) In terms of pharmacokinetics, D5 had four times higher activity than TX14A. D5Y levels in the brain peaked at 40–60 min and then reduced by 90 min 2) In NS20Y cells, D5Y had an ED50 of 0.16 nmol and the peak concentration in the brain was 4 nmol/g 3) D5 3 h after MCA occlusion had the lowest neurological score, followed by 6 h of MCA occlusion, and the saline control had the highest neurological score (i.e. the greatest post-ischemia behavioral deficit) 4) D5 reduced brain infarct area and volume, with 3 h post-occlusion having a stronger reduction than 6 h post-occlusion treatment
11113331	Lapchak et al.	2000	USA	In vivo: rabbits	Spinal cord ischemia	D5	Intravenous injection 5 min after start of occlusion (first set of experiments) or intravenous injection injection in normal rabbits (MAPK activity experiments)	1 mg/kg	Synthesized and purified by New England Peptide and was 98.7% pure by HPLC	1) D5 reduced the length of time to paraplegia in half the animals (ET50 measurement) compared to vehicle. Thus, prosaptide was detrimental as it reduced the tolerance to ischemia 2) D5 did not affect ERK phosphorylation in the spinal cord 3) D5 has active effects in the CNS up to 48 h after the single intravenous injection
11702044	Morita et al.	2001	Japan	In vitro: primary cortical neurons from E17 Wistar rats cerebral cortices (sex NA) in vivo: adult male Mongolian gerbils	Ischemia (3 min occlusion of common carotid arteries for 3 min)	PS18	Osmotic minipump implanted subcutaneously with a needle stretching out to the lateral ventricle	72 h after 3-min ischemia with 20 ng/day for 4 or 28 days (first series of studies) or for 4 weeks (second series of studies)	Unclear	1) PS18 prevents neuronal death in the hippocampus and ameliorated ischemia-induced learning disability (passive avoidance task) 2) PS18 increases the number of intact synapses and neuronal morphology in the hippocampus 3) PS18 prevents NO-induced toxicity and neuronal death in primary cortical neurons 4) PS18 increases the concentration of Bcl-xL mRNA in primary cortical neurons
38114054	Yu et al	2024	China	In vivo: 238 male and 18 female Sprague–Dawley rats (age NA)	Middle cerebral artery occlusion (induced by 4–0 nylon suture to occlude middle cerebral artery)	Recombinant PSAP	intranasally or intracerebroventricular injection	Experiment 2: 1, 3, or 9 ug/kg rPSAP Experiment 3: 9 ug/kg rPSAP Experiment 4: 9 ug/kg rPSAP Experiment 5: 9 ug/kg rPSAP	Unclear	1) PSAP and GPR37 were increased after ischemia with a peak at 24 h, but PSAP had significantly decreased by 72 h 2) 9 ug/kg rPSAP, but not 1 or 3 ug/kg, reduced infarct volume and neurological deficits (modified Garcia and beam walking scores) at 24 h post-MCAO 3) 9 ug/kg rPSAP improved performance on behavioral tests (rotarod test) 4 weeks post-MCAO 4) Intranasal rPSAP administration increased PSAP expression in the brain (ipsilateral hemisphere), and siRNA KD of PSAP increased neurological deficits post-MCAO 5) 24 h after MCAO, 9 ug/kg rPSAP elevated p-Akt, p-ASK1 and Bcl-2, but decreased p-JNK and Bax 6) 24 h after MCAO, rPSAP reduced neuronal apoptosis (FJC staining and TUNEL staining) by GPR37/PI3K/Akt/ASK1 mediated signaling
Synuclein										
11292674	Liu et al.	2001	USA	In vitro: primary culture dopaminergic neurons from ventral mesencephalic tissues of gestation from E15 Sprague–Dawley rats (sex NA) treated with MPTP in vivo: male 12-week-old C57BL/6 mice with MPTP-induced PD	Drug-induced PD	D1; D5	In vitro: 1, 5, or 10 ng/mL added directly to cell culture in vivo: subcutaneous injection with 50, 100, or 200 ug/kg every other day for 2 weeks (7 injections total)	D5: 50, 100, or 200 ug/kg in mice and 1, 5, or 10 ng/mL in cell culture; D1: 200 ug/kg in mice or 10 ng/mL in cell culture	Reverse-phase HPLC as described in [43], PMID: 10454138	1) 1, 5, and 10 ng/mL Prosaptide D5 rescued the number of primary neurites in primary DA neurons from 20 uM MPP + 2) 1 or 5 ng/mL Prosaptide D5 rescued the number of secondary neurites in primary DA neurons from 20 uM MPP+, and 10 ng/mL Prosaptide D5 not only rescued but also further increased the number of secondary neurites compared to vehicle 3) Prosaptide D5 dose-dependently increased primary DA neuron number 4) Prosaptide D5 dose-dependently rescued DA uptake in the presence of 20 uM MPP+, with 10 ng/mL completely rescuing DA uptake, an effect equivalent to 1 ng/mL GDNF 5) 25 uM of MAPK inhibitor PD98059 blocked Prosaptide D5's rescuing effect on DA neuron survival and neurite sprouting 6) Prosaptide D5 (subcutaneously administered as 50, 100, or 200 ug/kg) dose-dependently rescued DA neuron number in the substantia nigra in MPTP-treated mice, and 200 ug/kg D1, an inert all D-linear peptide, had no rescuing effect
23321539	Gao et al.	2013	Japan	In vivo: male 10-week old C57BL/6 J mice; in vitro: SH-SY5Y human neuroblastoma cells	Parkinson’s disease	PS18	In vivo: subcutaneous injection; in vitro: direct addition to cell culture	Mouse experimental group 1: 2 mg/kg PS18 once daily for 30 days Mouse experimental group 2: 2.0 mg/kg PS18 once daily for 5 days Mouse experimental group 3: 0.2 mg/kg, 2.0 mg/kg, or 10.0 mg/kg PS18 for 5 days Mouse experimental group 4: 2 mg/kg PS18 once daily for 30 days In vitro: 50, 100, 200, 300, 500, or 1000 ng/mL PS18	Synthesized by Operon Technology	1) 2 mg/kg PS18 administration had no long-term toxicity effects 2) In healthy mice, PS18 injection by itself did not alter TH expression level in the SNpc or striatum 3) 2 and 10 mg/kg PS18, but not 0.2 mg/kg, prevented loss of TH-positive neurons, though 10 mg/kg PS18 slightly decreased SNpc TH expression level 4) PS18 rescued mice from MPTP-induced behavioral deficits (open field test) 5) PS18 protected dopaminergic SNpc and striatum neurons from MPTP-induced degeneration, via inhibited astrocyte activation (GFAP immunostaining and western blot) and prevented decrease in TH protein (western blots) 6) PS18 inhibited activation of cleaved caspase-3, and reduced JNK1/2 phosphorylation and c-Jun activation in the MPTP mice (western blots) 7) PS18 decreased BAX expression and increased Bcl-2 and Bcl-2/BAX ratio in the MPTP-mice 8) PS18 was absorbed into SH-SY5Y cells and rescued them from cell death induced by MPP+ 9) PS18 rescued MPP+ -treated SH-SY5Y cells from caspase-3 activation, and likewise inhibited JNK and c-Jun phosphorylation while increasing Bcl-2 and Bcl-2/BAX ratio (western blots)
36152140	Kojima et al.	2022	UK	In vitro: SH-SY5Y	Parkinson’s disease	PSAP; Sap C; TX14(A)	PSAP plasmid was overexpressed in cells (transfection). Flow-through assay (vesicles): TX14(A) or Saposin C were incubated in presence of fresh lipid vesicles	TX14(A) of saposin C: 5 uM	TX14(A) was obtained from Synpeptide and confirmed by mass-spectrometry. Saposin C was expressed in *E. coli *DE3 cells and purified from cell pellets	1) Overexpressing PSAP in SH-SY5Y cells reduced a-synuclein levels (western blots) in a GCase activity independent manner, and PSAP-GFP was targeted to the lysosome (live-cell imaging) 2) PSAP-GFP colocalized with RAB5 and RAB7, indicating PSAP secretion into culture medium 3) PSAP overexpression promoted GCase activity without altering GCase expression levels 4) PSAP overexpression did not alter p62 (autophagy marker), but it did increase the autophagosome markers LC3-II and LC3-II/LC3-1 ratio (though these increases were less than what was observed in EGFP overexpression cells) 5) PSAP knockdown increased a-synuclein and decreased SNCA mRNA levels, suggesting feedback inhibition on SNCA transcription 6) Sap C removed a-synuclein from artificial glucosylceramide-enriched lipid vesicles at the lysosomal pH, but TX14(A) did not have this effect
37208379	Wu et al.	2023	Taiwan	In vitro: primary rat neurons from E15 ventral mesencephalon tissue of Sprague–Dawley rats and SH-SY5Y cells; in vivo: Unilateral 6-OHDA-induced striatum Parkinson’s model in Sprague–Dawley rats (age and sex NA)	Parkinson’s disease	PS18	In vitro: Addition to cell culture directly in vivo: Intracerebroventricular administration	In vitro: primary cell culture: 50, 100, 150, or 300 nM; SH-SY5Y: 50 nM in vivo: 2 mM/20 uL	Obtained from Elabscience	1) 50–300 nM PS18 rescued rat primary ventromesencephalic cell cultures from 100 uM 6-OHDA induced dopaminergic neuronal loss (TH immunoreactivity measurement) 2) 50 nM PS18 rescued rat primary ventromesencephalic cell cultures from 100 uM 6-OHDA, 500 nM Tg, and 1 mM methamphetamine-induced TUNEL activity increase 3) 50 nM PS18 attenuated ER stress (induced by 200 nM Tg or 100 uM OHDA) in SH-SY5Y cells, as measured by reduced GLuc-SERCaMP activity 4) Striatal 6-OHDA lesion upregulated PSAP in the striatum and nigra (qRT-PCR) 5) PS18 rescued 6-OHDA rats from behavioral deficits (measured by horizontal activity, total distance traveled, vertical activity and rotation) 6) In 6-OHDA rats, PS18 attenuated the loss of DAT and PSAP levels in the lesioned striatum 7) In 6-OHDA rats, PS18 significantly blunted the increase in ER stress markers PERK, ATF6, CHOP, and BiP, and nonsignificantly blunted the increase in ATF6 (western blots). Additionally, PS18 increased nigra TH immunoreactivity (IHC) and TH protein level (western blot) but did not alter striatal TH immunoreactivity
37726325	He et al.	2023	Sweden	In vivo: cPSAPDAT, unilateral 6-OHDA-induced striatum Parkinson’s model or wild-type C57BL/6 J mice Unilateral AAV-α-syn-induced Parkinson’s substantia nigra model in Sprague–Dawley rats	Parkinson’s disease	AAV-PSAP	Mice: Stereotaxic injection into the substantia nigra pars compacta Rats: Encapsulated cell biodelivery (ECB) devices containing ARPE-19 cells secreting PSAP, PSAP-PGRN, or controls were surgically grafted into the a unilateral striatum	AAV-PSAP delivered at 1 × 10 [12] gc/ml, and 1 uL of solution was injected at 0.2 uL/min for 5 min thrice. For encapsulated cell biodelivery, the device was implanted into the ipsilateral striatum	AAV-PSAP obtained from Vector Biolabs. Encapsulated cell biodelivery device generated with clonal APRE-19 cells that overexpressed PSAP, PGRN, and PSAP-PGRN	1) AAV-PSAP rescued motor deficits in cPSAPDAT mice by reversing α-syn-induced worsening in apomorphine rotation tests and restoring striatal and nigral TH and DAT expression. 2) AAV-PSAP reversed the elevated accumulation of phosphorylated α-syn (p-Ser129) in cPSAPDAT mice and reduced the presence of large proteinase K-resistant aggregates 3) AAV-PSAP failed to affect mild p-Ser129 α-syn accumulation in WT mice 4) AAV-PSAP protects against 6-OHDA-induced dopaminergic degeneration by reducing apomorphine-induced rotations and preserving TH and DAT levels in the striatum and substantia nigra of wild-type mice 5) ECB-PSAP and ECB-PSAP-PGRN preserved locomotion, reduced apomorphine-induced rotations, and protected against α-syn-induced dopaminergic degeneration in rats. 6) ECB-PSAP maintained striatal DAT and VMAT2 levels, demonstrating neuroprotective effects in rats
Diabetes										
10374753	Calcutt et al.	1999	USA	In vivo: adult female Sprague–Dawley rats (age NA)	Streptozotocin-induced diabetes	TX14(A)	Subcutaneous injection three times weekly	20–1000 ug/kg body weight	The peptide was obtained from Myelos Neurosciences and synthesized to 98% purity by AnaSpec	1) PSAP mRNA is elevated in sciatic nerve of 8-week diabetic rats (Northern blots) 2) TX14(A) slightly reduced the weight loss and hyperglycemia in diabetic rats 3) TX14(A) blunted the increase in thermal response latency in diabetic rats and lowered it back to control levels 4) TX14(A) partially rescued the loss in MNCV and SNCV in diabetic rats 5) 200 or 1000 ug/kg TX14(A) completely prevented the increase in hindpaw thermal response latency in diabetic rats 6) 1000 ug/kg TX14(A) reversed the loss in large myelinated axon frequency compared to untreated diabetic rats
11046216	Calcutt et al.	2000	USA	In vivo: adult female Sprague–Dawley rats (age NA)	Streptozotocin-induced diabetes	TX14(A)	Intraperitoneal or intrathecal injection either single or three times weekly	Multidose treatment: thrice weekly with 200 ug/kg (first experiment) or 1 mg/kg (second experiment) TX14(A); single-dose treatment: 1, 20, 200, or 1000 ug TX14(A)	The peptide was obtained from Myelos Neurosciences and synthesized to 98% purity by AnaSpec	1) Prolonged TX14 treatment of 200 ug/kg thrice weekly for 4 weeks reduces acute hyperalgesia in diabetic rats and reduced progressive SNCV slowing. A single 200 ug/kg i.p. injection to vehicle diabetic rats 30 min before the formalin test prevented hyperalgesia as well in phases Q and 2 2) TX14(A) thrice weekly of 1 mg/kg for 8 weeks suppressed the flinch response to 0.5% formalin injection into the paw, but this effect only lasted for 48 h after final injection and did not persist at the 72 h timepoint 3) single TX14(A) injection prevented formalin hyperalgesia but did not change thermal response latencies 4) TX14(A) reversed defects in tactile allodynia and paw thermal hyperalgesia in a dose-dependent manner, thus, TX14(A) is antihyperalgesic in diabetic rats
18596543	Jolivalt et al.	2008	USA	In vivo: adult female Sprague–Dawley rats (age NA)	Streptozotocin-induced diabetes and sciatic nerve crush in rats	TX14(A)	1 mg/kg TX14(A) was delivered thrice weekly for 8 weeks of streptozotocin-induced diabetes to evaluate PSAP protein levels. 4 weeks of treatment with TX14(A), 1 mg/kg i.p. delivered thrice weekly after nerve crush injury	1 mg/kg	Obtained from Myelos Neurosciences	1) PSAP protein level was reduced in endoneurial fluid of diabetic rats 1 and 9 days after sciatic nerve crush 2) PSAP protein level was higher in the proximal nerve segment of diabetic rats 9 days after transection, but was similar to the control rats 1 day after transection 3) TX14(A)-treated diabetic rats had reduced regeneration distance 3 days post-injury, but 7 days post-injury, the TX14(A)-treated diabetic rats had a greater regeneration distance than vehicle-treated diabetic rats 4) On day 3 but not day 7, TX14(A) reduced nerve regeneration distance in control rats 5) TX14(A) rescued the uninjured limb of diabetic rats from deficits in MNCV and SNCV slowing, thermal hypoalgesia and formalin-evoked hyperalgesia compared to vehicle-treated diabetic rats. However, this was not a full rescuing effect as the values for the phenotypes were still significantly different than control rats 6) TX14(A) attenuated the decline in EDL muscle weight compared to vehicle. TX14(A) also blunted the increase in mean axonal diameter in diabetic rats 7) TX14(A) sped up recovery of toe spread distance in control rats, but diabetic rats treated with TX14(A) did not have complete recovery of toe spread 8) TX14(A) treatment in diabetic rats did not alter sciatic nerve PSAP protein levels
11589426	Mizisin et al.	2001	USA	In vivo: adult female Sprague–Dawley rats (age NA)	Streptozotocin-induced diabetes or galactose-feeding	TX14(A)	Subcutaneous injection thrice weekly	1 mg/kg in 250 uL PBS	The peptide was obtained from Myelos Neurosciences and synthesized to 98% purity by AnaSpec	1) TX14(A)-treated diabetic rats had significantly higher MNCV and SNCV han untreated diabetic rats after 8 weeks 2) TX14(A) treatment initiated at 8 weeks in untreated diabetic rats rescued MNCV and SNCV by week 14, whereas rats treated during the first 8 weeks but then taken off treatment showed a significant decline in both measures by week 14 3) TX14(A) partially rescued MNCV and SNCV values in galactose-fed rats at 16 weeks without affecting body weight or plasma glucose concentration 4) TX14(A) rescued the decrease in mean axonal diameter in sciatic nerve, but not in the dorsal root, in galactose-fed rats 5) In galactose-fed rats, TX14(A) partially reversed the decrease in large myelinated fiber frequency and increase in small fiber frequency in the sciatic nerve, but not in the dorsal root 6) TX14(A) did not rescue deficits in myelin splitting and ballooning in dorsal and ventral roots
Allodynia/pain										
10643816	Yan et al.	2000	USA	In vitro: NS20Y cells or synaptosomes. in vivo: male Sprague–Dawley rats (age NA),	Seltzer rat model of neuropathic pain	PSAP, D5, and D5Y	Synaptosomes experiments: incubation of prosaptides with synaptosomes. Pharmacokinetics experiment: intramuscular injection for 60 min; thermal hyperalgesia experiment: subcutaneous injection on day 20 post surgery	Synaptosomes binding experiment: 0, 1, 2, 5, or 10 nM D5. Pharmacokinetics experiment: 200 ug/kg [125I]D5Y intramuscular for 60 min; thermal hyperalgesia experiment: subcutaneous injection of 200 ug/kg D5 on day 20 post surgery. Synaptosomes Ca2+ uptake experiment: 2.5 nM PSAP, 8 nM D5	Synthesized and purified by reverse phase HPLC	1) D5 caused neurite growth in NS20Y with an ED50 of 0.16 nM, which was more potent than TX14(A) which had an ED50 of 0.62 nM 2) D5 increased [35S]GTPγS binding of synaptosomal membranes in a dose-dependent fashion 3) D5 reversed thermal hyperalgesia after 3 h of treatment 4) PSAP and D5, in a dose-dependent fashion, inhibited Ca2+ uptake. This effect was mediated by voltage-dependent calcium channels and a pertussis-sensitive GPCR. The mechanism for D5 alleviating hyperalgesia is via this pertussis-sensitive GPCR
16480831	Jolivalt et al.	2006	USA	Various metabolic, physical, neurotic, inflammatory damage models of induced allodynia in female Sprague–Dawley rats (e.g., diabetes, sciatic nerve hemiligation, systemic paclitaxel, paw formalin injection) (age NA)	Allodynia	TX14(A)	Dose–response intraperitoneal (0.02, 0.2, 0.4 mg/kg); for time course experiment TX14(A) 500 ug in 10 ul volume in diabetic rats	Dose–response on diabetes-induced tactile allodynia: 0.02, 0.2, 0.4, 1, or 4 mg/kg i.p. Time course: 1 mg systemic or 500 ug intrathecal; tactile allodynia: 4 mg/kg i.p., paclitaxel-induced allodynia: 4 mg/kg i.p. single dose, or 1 mg/kg given on alternate days from day 8 to day 24. Formalin-induced secondary allodynia: 4 mg/kg i.p. single dose or 1 mg/kg i.p. on days 1–5	Obtained from Myelos Corp	1) TX14(A) when administered systemically reduced allodynia in the partial sciatic nerve ligation model, rescued mice from formalin-induced secondary allodynia, paclitaxel-induced tactile allodynia, also had an anti-allodynic effect in diabetes-induced tactile allodynia mice, but this effect was weakened 24–48 h post-delivery
17466547	Jolivalt et al.	2008	USA	In vivo: adult female Sprague–Dawley rats	Tactile allodynia induced by gp120	TX14(A)	Prevention experiments: intraperitoneal, intraplantar, or intrathecal 0 min before gp120. Reversal: 70 min after gp120. TNFa experiments: intraperitoneal (i.p.)	Prevention experiments: 0.01, 0.1, or 4 mg/kg systemically delivered, intraplantar: 1 mg, intrathecal 0.5 mg. Reversal experiments: intraplantar 4 mg/kg, intrathecal 0.5 mg. TNFa experiments: 4 mg/kg i.p.	Obtained from Myelos Corp	1) TX14(A) protected rats from gp120 tactile allodynia in a dose-dependent manner (0.1 or 4 mg/kg had protective effect, but 0.01 mg/kg did not) 2) 4 mg/kg TX14(A) rescued mice from established tactile allodynia within 50 min post-injection, but 1 mg intraplantar administration of TX14(A) had no rescuing effect while 0.5 mg intrathecal TX14(A) 30 min before gp120 protected against allodynia for up to 6 h 3) 0.5 mg intrathecal TX14(A) given 70 min after gp120 injection rescued mice from tactile allodynia 4) TNFa levels were unaltered in the nerve and spinal cord of intraplantar gp120-injected rats. TX14(A) increased TNFa protein levels in the nerve compared to vehicle-treated gp120-injected animals
Drug-induced toxicity										
9553960	O’Brien et al.	1998	USA	In vitro: PC12 and Schwann cells in vivo: adult rats	Paclitaxel neurotoxicity	TX14(A) or 769P	Addition to cell culture directly	In vitro: 10 nM TX14(A), in vivo: 3 or 10 mg/kg prosaptides	N/A	1) TX14A rescued paclitaxel-induced cell viability loss in PC12 but not Schwann cells 2) 3 or 10 mg/kg prosaptide co-administration with paclitaxel in mice prevented thermal hypoalgesia. However, erythrocyte counts, hematocrit, and hemoglobin were not rescued 3) TX14(A) did not reduce cytotoxicity of paclitaxel against breast cancer cells in vitro, suggesting TX14(A) is neuroprotective without altering anti-tumor effects of paclitaxel
AB/Tau										
27372641	Gao et al.	2016	China	In vivo: male 12-week old C57BL/6 J mice	AD	PS18	Subcutaneous injection	0.2 mg/kg or 2 mg/kg PS18 or vehicle once a day for 7 consecutive days post-amyloid beta injection	Synthesized by Operon Technology	1) PS18 rescued mice from Ab-induced memory impairment (measured by Morris water maze). 2 mg/kg PS18 had a stronger therapeutic effect than 0.2 mg/kg, as 0.2 mg/kg did not alter passing times in the Morris water maze compared to Ab mice 2) 2 mg/kg but not 0.2 mg/kg PS18 increased hippocampal Ach, cortex Ach, hippocampal ChAT activity, cortex ChAT activity, and ChAT protein expression levels 3) PS18 significantly increased cell migration and differentiation as measured by PSA-NCAM positive cells in the granular zone. PS18 also increased neurogenesis (BrdU-labeled cells) compared to untreated Ab mice 4) PS18 reduced expression of neuroinflammatory markers Iba-1 and GFAP (western blots) in the dentate gyrus 5) PS18 blunted BACE-1 overexpression in Ab mice, and reduced PS1 and PS2 compared to untreated AB mice while increasing disintegrin and ADAM10 expression (western blots). PS18 also corrected Ab-induced tau hyperphosphorylation on Ser199/202 and Ser396 compared to untreated Ab mice 6) PS18 increased hippocampal p-PDK1, increased Ser473 Akt phosphorylation, and prevented loss of GSK-3β_x0002_ (Ser 9) phosphorylation compared to untreated Ab mice (western blots) 7) PS18 blunted the Ab-induced activation of caspase-9 in hippocampi, reduced active caspase-3 and caspase-3 levels. It also decreased PARP-1 levels and BAX expression but increased Bcl-2 expression while correcting the Bcl-2/BAX ratio. Overall, PS18 corrected many of the molecular deficits in the Ab-treated mice

It is important to note that the focus on periphery-CNS communication has not focused on PSAP. This is because PSAP is unlikely to be a viable molecule for effective blood-to-brain delivery due to its large size, extensive glycosylation, and intracellular routing. Although PSAP is secreted and detectable at relatively high concentrations in circulation, its primary intracellular fate is lysosomal, where it is processed into saposins A through D that function as lipid-hydrolyzing cofactors. These domains remain in the lysosome to support sphingolipid metabolism and are not thought to act independently as blood-borne signaling molecules. Furthermore, the full-length PSAP protein lacks features commonly associated with efficient BBB transit, such as specific receptor-mediated transport mechanisms or structural properties enabling passive diffusion. Its physicochemical properties and lysosomal routing suggest that despite its presence in the periphery, PSAP is poorly suited for CNS delivery without specialized transport systems. For these reasons, motivated researchers aimed to develop recombinant fragments containing portions of this sequence, collectively termed “prosaptides.”

To investigate peripheral-to-brain delivery of prosaptide derivatives, one of the earlier studies synthesized five retro-inverso peptide mimetics in which the amino acid sequence is reversed and is composed of D-amino acids instead of the native L-forms. This often leads to stabilization as it renders the de novo peptides less susceptible to enzymatic modifications or degradation. These five retro-inverse prosaptides were compared to previously studied L-amino acid prosaptides TX14(A) and TX15-2 (TXI^*^IDNNKTEKEISY), which have been shown to be rapidly cleared from the circulation and brain [191]. The authors radiolabeled the peptides with iodine-125 and injected them intravenously into male rats and collected serum and brain tissue at various time points. Despite showing modest evidence for retro-inverso prosaptide D4 (YLL^*^E-ETANNDL^*^) crossing the blood–brain barrier by passive diffusion (*K*_*i*_ = 2.5 × 10^–4^ ml·g^−1^. min^−1^), its transfer rate was ~ tenfold lower than TX14(A) and TX15-2, limiting its CNS therapeutic potential ^192^.

Following the initial interest in D4, the authors suddenly shifted toward testing D5 through peripheral injections in various in vivo models, including ischemia [193], hyperalgesia [50], and myelination [193], reporting improvements in each. However, D5 was reported to worsen outcomes in a rabbit spinal cord ischemia model, where it was administered intravenously at 1 mg/kg 5 min after the onset of aortic occlusion. While vehicle-treated rabbits showed expected recovery profiles based on occlusion time, those treated with D5 exhibited earlier onset of paraplegia [41]. Retro-inverso peptide mimetic testing ceased shortly after this for unknown reasons. Attention then shifted to PS18, a commercially available prosaptide shown to exert neuroprotective and regenerative effects when administered in vitro or delivered directly into the CNS *in vivo*[36, 37, 42, 57]. PS18 peripheral-CNS effects were also tested in vivo across kainic acid-induced injury [45], AD [53], PD [56], neuronal apoptosis [38], and healthy models [188], suggesting modest CNS effects.

However, despite reported therapeutic effects, studies examining peripheral administration of the prosaptide analogues D5 and PS18 exhibit major deficiencies in both pharmacokinetic and pharmacodynamic evaluations. Critically, none of the available studies demonstrate that these peptides reach the brain following systemic injection, raising the possibility that observed effects are mediated by peripheral rather than central mechanisms. Fundamental pharmacokinetic parameters, including plasma clearance, enzymatic stability, biodistribution, and central nervous system accumulation, were not assessed. No study employed radiolabeling, peptide tagging, or mass spectrometry to trace localization, nor did any investigate time-dependent concentration profiles in plasma or brain tissue. For instance, one study administered PS18 intraperitoneally at a high dose of 2.0 mg/kg in healthy male mice and reported upregulation of only two synaptic proteins in the highly enriched synaptic markers region, the “synaptosome” [188]. Moreover, many of the studies had a small sample size, lacked technical validation, and had not been independently replicated, some for even decades.

In the absence of rigorous pharmacokinetic characterization and reproducible pharmacodynamic data, it remains highly uncertain whether systemically administered prosaptides can exert direct effects within the CNS. It is likely that peripheral delivery alone is insufficient and that enhanced strategies such as nanoparticle encapsulation or EV-based delivery may be required to facilitate brain penetration to a pharmacodynamically relevant concentration sufficient to elicit a physiological effect [194, 195].

## PSAP as a therapeutic target in clinical trials

Following PSAP’s and prosaptides’s preclinical efficacy in models of allodynia [51], diabetic neuropathy [46, 47], and nerve injury [43, 196], the 14-mer derivative (TX14(A)) was used to treat diabetic-associated neuropathy and later HIV-associated neuropathy in humans. TX14(A) was tested in a phase 2 randomized placebo-controlled trial in persons with type 1 or 2 diabetic neuropathy (FDA IND number 66074, *n* = 150), self-administered subcutaneously at doses of 1, 4, or 16 mg per day for 28 days. Although results were never formally published, a secondary report [197] suggested pain reduction in the 4 mg group but noted diminished efficacy in severe neuropathies. A subsequent phase 2 trial (clinicaltrials.gov ID: NCT00286377) in HIV-associated sensory neuropathy (*n* = 237) tested daily doses of 2–16 mg for 6 weeks but failed to show significant differences across treatment arms, leading to early termination after futility analysis [197]. Given that TX14(A) was shown to be rapidly cleared from the rat blood and brain [191] and that no detailed pharmacokinetic and pharmacodynamic studies have been performed on it, this is not unexpected. Following these trials, clinical development of prosaptides was discontinued, but they remain commercially available for laboratory experimentation.

No current therapeutics exist for specifically targeting PSAP. However, therapeutics targeting PSAP-PRGN complexes have been tested in tauopathies such as AD (preclinical) and PSP (clinical). AZP2006, an orally available small molecule, augments PRGN’s half-life by stabilizing PSAP-PRGN complexes, leading to reduced PRGN-related degradation into inactive peptides and prolonging PRGN’s neuroprotective properties [198] to enhance lysosomal health and reduce tau pathology. In AD mouse models, AZP2006 reduced tau hyperphosphorylation and neuroinflammation and promoted neuronal survival and synaptogenesis [199].

The phase 1 study of AZP2006 was conducted in healthy volunteers and consisted of single ascending dose (*n* = 64) and multiple ascending dose (*n* = 40) arms to evaluate its safety, tolerability, and pharmacokinetics. The drug showed a favorable safety profile, was well tolerated across dose ranges, and demonstrated rapid absorption with predictable, multiphasic elimination [200]. Following the successful phase 1 trial, AZP2006 advanced to a phase 2a randomized, double-blind, placebo-controlled study (clinicaltrials.gov ID: NCT04008355) in persons with PSP. Over 12 weeks, AZP2006 was administered in two dosing regimens: 60 mg once daily or 80 mg for 10 days followed by 50 mg daily. The drug maintained its favorable safety profile with no significant adverse effects. Although the primary efficacy endpoint, the PSP Rating Scale, did not show a statistically significant difference between treatment and placebo, some secondary clinical measures suggested potential benefit. These findings, combined with good tolerability, supported continued clinical development in a larger phase 2b/3 trial to assess its long-term efficacy [59].

## Conclusion

Throughout the past three decades, PSAP (and its prosaptide derivatives) have been studied across diverse experimental systems in CNS health and disease. Early studies revealed its strong neurotherapeutic and neuroprotective effects and linked PSAP genetic variants to multiple neurodegenerative disorders. In recent years, prosaptides have gained renewed attention in models of AD and PD, showing preserved synaptic integrity, behavioral improvements, and modulation of disease-relevant molecular pathways. However, emerging evidence suggests PSAP may play dual roles, exerting protective effects in some contexts while contributing to tumorigenesis, neuroinflammation, and neurotoxicity in others, including brain cancers. PSAP expression is also highly responsive to various forms of metabolic stress, such as cold adaptation and PGC1α-driven remodeling, yet remains unaltered or downregulated by acute and chronic exercise, indicating it is not an exerkine or an exercise-responsive signaling molecule. Despite promising preclinical data, translation into human studies remains limited. Future studies should focus on enhancing the pharmacokinetics of PSAP and its prosaptides, such as through EV or nanoparticle-based delivery systems, and further explore the utility of PSAP as a biomarker for neurodegenerative disorders, especially given the current scarcity of human data.

## Supplementary Information

Below is the link to the electronic supplementary material.

ESM 1 (XLSX 189 KB)

## Data Availability

Any data analyzed is included in the article.
